# Exploring the Wnt Pathway as a Therapeutic Target for Prostate Cancer

**DOI:** 10.3390/biom12020309

**Published:** 2022-02-15

**Authors:** Sarah Koushyar, Valerie S. Meniel, Toby J. Phesse, Helen B. Pearson

**Affiliations:** 1 The European Cancer Stem Cell Research Institute, School of Biosciences, Cardiff University, Hadyn Ellis Building, Cardiff CF24 4HQ, UK; S.Koushyar@kingston.ac.uk (S.K.); MenielVS@cardiff.ac.uk (V.S.M.); 2 School of Life Sciences, Pharmacy and Chemistry, Faculty of Science, Engineering and Computing, Kingston University, Penrhyn Road, Kingston Upon Thames KT1 2EE, UK; 3 The Peter Doherty Institute for Infection and Immunity, The University of Melbourne, Melbourne 3000, Australia

**Keywords:** *APC*, β-catenin, CRPC, metastasis, prostate cancer, targeted therapy, Wnt

## Abstract

Aberrant activation of the Wnt pathway is emerging as a frequent event during prostate cancer that can facilitate tumor formation, progression, and therapeutic resistance. Recent discoveries indicate that targeting the Wnt pathway to treat prostate cancer may be efficacious. However, the functional consequence of activating the Wnt pathway during the different stages of prostate cancer progression remains unclear. Preclinical work investigating the efficacy of targeting Wnt signaling for the treatment of prostate cancer, both in primary and metastatic lesions, and improving our molecular understanding of treatment responses is crucial to identifying effective treatment strategies and biomarkers that help guide treatment decisions and improve patient care. In this review, we outline the type of genetic alterations that lead to activated Wnt signaling in prostate cancer, highlight the range of laboratory models used to study the role of Wnt genetic drivers in prostate cancer, and discuss new mechanistic insights into how the Wnt cascade facilitates prostate cancer growth, metastasis, and drug resistance.

## 1. Introduction

Prostate cancer is the second most common cancer in men, with global prostate cancer-related deaths exceeding 375,000 annually [1]. Patients diagnosed with localized or regional disease generally have a favorable outcome (5-year survival > 99%). However, survival outcome is considerably reduced for patients with distant metastatic prostate cancer (5-year survival < 30%) [2]. Metastatic prostate cancer can be present either at the time of diagnosis, termed hormone-sensitive (mHSPC), or following therapeutic resistance to androgen/androgen receptor (AR) pathway inhibitors, known as metastatic castration-resistant prostate cancer (mCRPC) [3]. 

Over the past decade, phase III clinical trials exploring new treatment options for mHSPC have led to a shift in the standard of care from androgen deprivation therapy (ADT) alone to ADT in combination with taxane-based chemotherapy (CHAARTED, GETUG-AFU 15, STAMPEDE), or novel androgen/AR-targeted therapies such as abiraterone acetate (STAMPEDE, LATITUDE), enzalutamide (ARCHES, ENZAMET) and apalutamide (TITAN) [4,5,6,7,8,9,10]. For example, the CHAARTED trial revealed that docetaxel treatment in combination with ADT improved the overall median survival by 13.6 months compared to ADT alone (hazard ratio (HR) = 0.61, 95% confidence interval 0.47–0.80) [4], while patients receiving enzalutamide in the ENZAMET trial displayed increased overall survival at 36 months relative to non-steroidal anti-androgens (HR = 0.67, 95% confidence interval 0.52–0.86, initial interim findings) [7]. Of note, treatment discontinuation in the enzalutamide treatment arm was higher than the standard of care arm in the ENZAMET trial, reflecting adverse side effects (e.g., fatigue, seizures, and periphery neuropathy), especially in patients treated early with docetaxel [7], and new approaches to improve the clinical safety profile for this patient group are currently being explored. 

Standard treatment regimens for mCRPC patients include ADT, sipuleucel-T, chemotherapeutic agents (e.g., docetaxel and cabazitaxel), abiraterone acetate, enzalutamide, and radium-223 for bone metastasis. In 2020, poly (ADP-ribose) polymerase (PARP) inhibitors (e.g., olaparib and rucaparib) were also approved by the FDA for the treatment of metastatic prostate cancer harboring inactivating mutations in key DNA damage repair genes, such as *BRCA1* and *BRCA2*, following the success of the PROfound and TRITON trials [11,12,13]. However, despite the evolving range of treatment options for mHSPC and mCRPC, disease progression is inevitable, and survival rates remain low as the aggressive cancer cells instigate new molecular mechanisms to evade treatment. Thus, there is an urgent unmet clinical need to develop new therapeutic strategies that can either prevent or treat metastasis to improve patient health. 

The Wnt signaling cascade is an evolutionarily conserved pathway that plays a key role in regulating multiple cellular events during embryonic development and normal adult tissue homeostasis. These include stem cell function, proliferation, survival, motility, and differentiation [14]. Deregulation of Wnt signaling has been functionally demonstrated to promote many aspects of cancer, including tumor initiation, progression, dissemination, and treatment resistance [15,16,17]. Wnt signaling is commonly activated in prostate cancer, particularly metastatic prostate cancer, and clinical data has revealed oncogenic Wnt signaling is correlated with a high Gleason score, high prostate specific antigen (PSA) serum levels, early disease onset (<50 years of age), and disease recurrence [15,16,18,19]. Thus, these findings indicate that the Wnt pathway presents a novel therapeutic target for prostate cancer. Accordingly, several inhibitors suppressing Wnt signaling have been developed, and preclinical studies have begun to explore their efficacy in prostate cancer [18,19,20,21,22,23,24].

In addition to mediating downstream effector cascades that promote cancer growth and metastatic spread, the Wnt pathway has also been shown to cooperate with other cell signaling pathways to facilitate prostate cancer and CRPC transition, including the AR pathway [25,26,27,28]. Consequently, a deeper understanding of the molecular mechanisms underpinning Wnt-driven prostate cancer and CRPC is paramount for the discovery of predictive biomarkers and effective therapeutic approaches targeting this pathway. To this end, carefully designed preclinical studies to establish which patient cohort is likely to respond to Wnt-targeted therapies, and whether Wnt inhibition is sufficient to inhibit or prevent metastasis, are required to help inform treatment decisions to benefit prostate cancer patients.

### 1.1. Wnt Signaling Pathway

**Figure 1:**
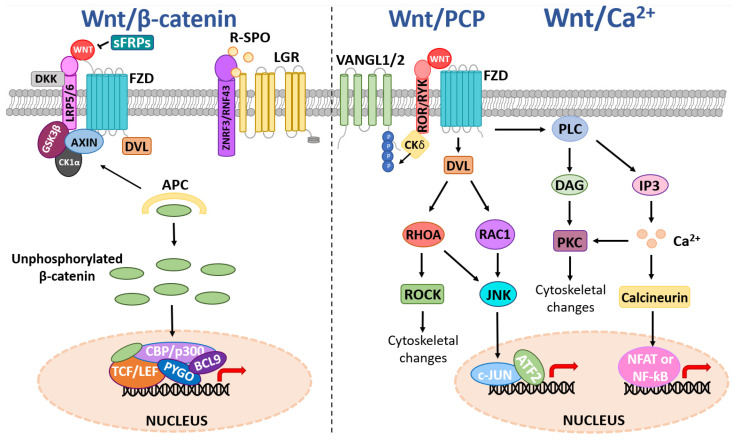
Schematic of canonical and non-canonical Wnt signaling. Canonical Wnt signaling is activated upon Wnt ligand binding to FZD Wnt receptors and Wnt co-receptors such as *LRP5*/*6*, causing the recruitment of DVL to the plasma membrane. This causes the destruction complex (AXIN1/2, *APC*, GSK3β, CK1α) to dissociate, allowing the stabilization and accumulation of unphosphorylated β-catenin in the cytoplasm. β-catenin can then translocate to the nucleus where it associates with TCF/LEF, CBP/p300, PYGO1/2, and *BCL9* to regulate the expression of Wnt target genes. Negative regulation of the canonical Wnt pathway can occur via multiple mechanisms, including extracellular sFRPs preventing FZD-Wnt binding and DKK-mediated inhibition of *LRP5*/*6*. Wnt ligand activation of the non-canonical Wnt/PCP pathway involves signal transduction via a complex of FZD Wnt receptors and Wnt co-receptors (e.g., ROR, RYK, and VANGL1/2), leading to plasma membrane recruitment and activation of DVL and CK1δ/ε-mediated phosphorylation of VANGL2. DVL binds to the small GTPase; Rac1, RhoA to activate ROCK and JNK. This can lead to ROCK-mediated cytoskeletal rearrangements or JNK regulation of target genes via phosphorylation of transcription factors such as c-JUN, which can associate with proteins such as activation of transcriptional factor 2 (ATF2). The Wnt/Ca^2+^ pathway activation leads to increased PLC activity, stimulating the production of DAG that activates PKC, and IP3 that triggers intracellular release of Ca^2+^ ions. This results in downstream signaling events such as cell cytoskeletal rearrangements and calcineurin-mediated transcriptional responses via transcription factors such as NFAT or NF-κB.

The Wnt family is comprised of 19 secreted glycoproteins (encoded by *WNT-1*, *2*, *2B*, *3*, *3A*, *4*, *5A*, *5B*, *6*, *7A*, *7B*, *8A*, *8B*, *9A*, *9B*, *10A*, *10B*, *11*, and *16*) that transmit intracellular signals to activate β-catenin dependent (canonical) or β-catenin independent (non-canonical) signaling events by binding with varying affinities to the Wnt receptor complex at the plasma membrane, reviewed in [29,30]. Frizzled (FZD1-10) Wnt receptors can homo-polymerize and associate with various Wnt co-receptors, including low-density lipoprotein receptor-5/6 (*LRP5*/*6*), receptor tyrosine kinase-like orphan receptor 2 (ROR2), and receptor-like tyrosine kinase (RYK), which further convolute the Wnt cascade [29,30,31,32]. In an off-state, canonical Wnt signaling is suppressed by ubiquitin-directed degradation of β-catenin, which is mediated through β-catenin phosphorylation events coordinated by the β-catenin destruction complex (Figure 1) [30,31,33,34]. The destruction complex is composed of scaffold proteins, including axis inhibitor 1/2 (AXIN1/2), the tumor suppressor adenomatous polyposis coli (*APC*), and two kinases that phosphorylate β-catenin, casein kinase 1α (CK1α) and glycogen synthase-3 beta (GSK3β) [29,30,31,32,33]. In an on-state, canonical Wnt signaling is activated by Wnt ligands binding to the FZD receptor via a palmitoleic acid lipid group and hydrophobic amino acid contacts [34]. Wnt-bound FZD can then form a complex with co-receptors *LRP5*/*6*, causing receptor polymerization that allows the recruitment of disheveled (DVL) and AXIN to the cell membrane thus the destruction complex can no longer phosphorylate β-catenin [31,32]. Subsequently, β-catenin can accumulate and translocate to the nucleus, where it associates with members of the T-cell factor/lymphoid enhancer factor (TCF/LEF) family to form a transcriptional activation complex with CREB binding protein (CBP), p300, pygopus family PHD finger 1/2 (PYGO1/2) and B-cell lymphoma 9 (*BCL9*) to regulate expression of Wnt target genes. Examples of Wnt target genes include *CCND1* (encoding CyclinD1), which regulates the G1 phase of the cell cycle, and *MYC*, a proto-oncogene and transcription factor that coordinates S phase of the cell cycle enabling cell cycle progression [35]. 

There is considerable regulation of the Wnt pathway at the level of the receptor/ligand. The turnover of FZD Wnt receptors on the cell membrane is controlled by the E3-ubiquitin ligases ring finger protein 43 (RNF43) and zinc and ring finger 3 (ZNRF3) (Figure 1) [31]. In turn, RNF43/ZNRF3 are cleared from the plasma membrane when R-Spondin (RSPO) binds to Leucine Rich Repeat Containing G Protein-Coupled Receptor 4/5/6 (*LGR4*/*5*/*6*) and forms a complex with RNF43/ZNRF3, thus allowing the FZD receptor and Wnt ligand to interact and activate Wnt signaling. Dickkopf Wnt signaling pathway inhibitor 1/2/4 (DKK1/2/4) binds and inhibits *LRP5*/*6* to block Wnt signaling, whilst secreted frizzled-related proteins (sFRPs) antagonize Wnt signaling through direct interactions with Wnt ligands and FZD receptors [31].

The non-canonical Wnt signaling pathway is β-catenin independent and can be broadly categorized into three pathways; the planar cell polarity (PCP) pathway, the Wnt/Ca^2+^ pathway, and the Wnt/STOP pathway, reviewed in [30,31,36]. Activation of the PCP pathway by Wnt ligands (e.g., Wnt5A and Wnt11) binding to Wnt receptors/co-receptor complexes potentiates small GTPases including ras homolog family member A (RhoA) and rac family small GTPase substrate (Rac), and subsequent downstream signaling events such as jun-N-terminal kinase (JNK), Rho kinase (ROCK) and activating transcription factor 2 (ATF2) signaling to regulate cellular processes, including cell polarity, differentiation, proliferation and motility [36,37,38,39,40,41,42] (Figure 1). Wnt ligands can also bind to Wnt co-receptors such as RYK, ROR1/2, and van gogh-like protein 1/2 (VANGL1/2). Upon Wnt binding, ROR2 can also form a receptor complex with VANGL2 leading to subsequent phosphorylation of VANGL2 by casein kinase 1 delta/epsilon (CK1δ/ε) [40]. VANGL2 has no known receptor or enzyme activity but is considered to play a scaffolding role to modulate downstream PCP signaling activity, including RhoA and Rac1, via protein-protein interactions [36]. Activation of the Wnt/Ca^2+^ pathway increases intracellular signaling molecules 1,2 diacylglycerol (DAG) and inositol 1,4,5-triphosphate (IP3) via phospholipase C (PLC). This, in turn, activates protein kinase C (PKC) and triggers intracellular Ca^2+^ fluxes that potentiate calmodulin and/or PKC to mediate cytoskeletal rearrangements and transcriptional activation of target genes via the nuclear factor of activated T-cells (NFAT) or nuclear factor kappa B (NF-κB) [43,44]. The more recently discovered non-canonical Wnt/STOP pathway involves Wnt/LRP6-mediated, β-catenin-independent stabilization of proteins involved in cellular division via Cyclin Y and remains to be fully characterized [45,46]. 

### 1.2. Wnt Signaling and AR Crosstalk

Despite initial responses to ADT, prostate cancer cells eventually develop genetic variants and/or instigate therapeutic resistance mechanisms to overcome treatment and promote CRPC growth and uncurable metastatic disease (reviewed in [28,29,47]). Genetic drivers of CRPC include those that trigger AR-dependent resistant growth, such as *AR* amplification associated with AR hypersensitivity, and *AR* mutations that increase AR transactivation by reducing ligand specificity [26,48]. Reduced ligand specificity enables AR activation via steroid hormones other than androgens (e.g., progesterone, estrogen and glucocorticoids), or adrenal androgens that are not targeted by ADTs [26]. *AR* splice variants that lack a ligand-binding domain (LBD) are also augmented in CRPC patients (e.g., *AR-V7*), which render LBD-targeting AR inhibitors such as bicalutamide and enzalutamide ineffective [48]. Alternatively, AR bypass signaling can arise via glucocorticoid receptor (GR) or progesterone receptor (PR) activation, which can regulate AR target genes [49]. Crosstalk between the AR pathway and interacting signaling cascades such as the PI3K (Phosphoinositide 3-kinase), MAPK (Mitogen-activated protein kinase) and Wnt pathways has also been shown to facilitate CRPC [19,28,29,47,50]. This highlights the diversity of the molecular mechanisms underpinning castration-resistance and emphasizes the need for novel approaches to detect and/or prevent the emergence of CRPC and the discovery of effective CRPC treatments. 

Interactions between the Wnt and AR pathway have been extensively studied, revealing the Wnt pathway can regulate AR signaling via multiple mechanisms. For instance, Wnt3a has previously been shown to enhance AR-mediated transcription in LNCaP prostate cancer cells in vitro, leading to enhanced cell growth and colony formation [51]. Furthermore, β-catenin is reported to directly bind the LBD of AR in a dihydrotestosterone (DHT)-dependent manner via its armadillo domain, resulting in increased AR-mediated transcriptional activity in AR-positive LNCaP prostate cancer cells [52]. Several studies have also reported that canonical and non-canonical Wnt signaling may contribute to CRPC growth [47,50,53,54,55,56]. The mechanistic basis for the Wnt pathway facilitating CRPC growth is currently unclear. However, recent studies have shown Wnt signaling can activate AR-mediated transcription by augmenting yes-associated protein (YAP, a key mediator of the Hippo pathway) to promote androgen-independent cell proliferation [56], while androgen deprivation has been shown to increase β-catenin:TCF4 interactions [55]. The Wnt ligand secretion mediator wntless (WLS) has also been shown to confer enzalutamide resistance in prostate cancer cells [57], and Wnt target genes *LEF1* and *WNT5a* are elevated in LNCaP-AI cells (cultured in androgen-depleted media) [47]. AR can also bind to androgen response elements (AREs) in the promoter regions of *LEF1* and *WNT5a* [47]. Interestingly, knockdown of *LEF1* and *WNT5a* is reported to suppress androgen-independent, but not androgen-dependent cell proliferation [47]. Together, these data suggest that a deeper understanding of AR signaling and Wnt pathway interactions may be instrumental in identifying new therapeutic approaches to prevent and/or treat CRPC. 

Emerging evidence has revealed that Androgen/AR-targeted therapies are associated with treatment-emergent neuroendocrine prostate cancer (t-NEPC), an aggressive form of CPRC where tumors gain the ability to transdifferentiate from adenocarcinoma to a neuroendocrine subtype [28,58,59,60]. Although the molecular mechanisms for t-NEPC are largely unknown, WLS, which facilitates Wnt secretion, has recently been discovered as a key driver of t-NEPC [24]. Using an in vitro model of enzalutamide-induced NEPC (C4-2B^ENZR^) and clinical specimens, AR pathway inhibitors have been shown to be associated with increased canonical/non-canonical Wnt signaling and the alleviation of AR-mediated transcriptional suppression of *WLS*. This resulted in elevated WLS and neuroendocrine markers in CRPC and NEPC patient samples and a reduction in androgen-responsive genes [24]. Mechanistically, WLS was found to mediate ROR2-PKCδ-ERK signaling to facilitate NEPC growth [24]. In support of a role for aberrant Wnt signaling during t-NEPC, TCF4 has also been shown to induce enzalutamide resistance via neuroendocrine differentiation in prostate cancer [61], and interestingly, FOXB2-Wnt7B signaling and Wnt11 are also reported to induce neuroendocrine differentiation and neuroendocrine marker expression in prostate cancer cells [62,63]. Together, these findings indicate the Wnt cascade is an attractive therapeutic target for prostate cancer. 

### 1.3. Wnt Pathway Genetic Alterations in Prostate Cancer 

Large-scale genomic sequencing and gene expression profiling studies of clinical prostate cancer specimens have identified a variety of genetic alterations in Wnt signaling pathway components that can promote tumor progression, treatment resistance, and metastasis [16,28,64,65,66,67]. To deepen our understanding of the frequency of Wnt pathway genetic alterations in primary and metastatic prostate cancer, we used the cBioPortal platform to survey three publicly available prostate cancer genomic datasets with mutation and copy number variation data for a panel of 70 genes that encode key Wnt cascade components/effectors [68,69]. OncoPrints of the genetic alterations observed highlight a wide variety of Wnt pathway genetic alterations in prostate cancer, particularly metastatic prostate cancer, and illustrate several alterations that tend to co-occur (Figures S1–S4 and Tables S1–S4). Moreover, genetic alterations were observed in multiple components of the Wnt pathway, including Wnt receptors, Wnt co-receptors, extracellular regulators, and intracellular signaling components (Tables S1–S4). The most common variants are summarized in Table 1.

While mutations in the Wnt pathway genes assessed are generally rare in primary prostate cancer (≤1%), inactivation mutations in *APC* (primary: 1.6–2.7% incidence, metastatic: 6.3–7%) and activating mutations in *CTNNB1* (Catenin Beta 1) that encodes β-catenin (primary: 1.8–2.6% incidence, metastatic: 4.3–5.4%) are relatively common, particularly in metastatic prostate cancer (Table 1). Copy number variation analysis revealed frequent amplification of several Wnt receptors/co-receptors (*FZD6*, *FZD9*, *LGR6*, *LRP5*, and *RYK)*, extracellular regulators (*RSPO2*, *DKK4*, and *SFRP1*) and intracellular components (*BCL9*, *DVL3*, and *PYGO2*), and homozygous deletion of *FZD3* and *APC* were also common. While significant evidence in the literature has identified *APC* loss (truncation mutation or homozygous deletion) and *CTNNB1* activating mutations stimulate Wnt signaling, the functional consequence of the other genetic variants remains unclear and warrants further investigation. This will require in-depth functional analysis of Wnt pathway activity in response to these genetic alterations, both alone and in combination with other genetic aberrations that have a strong tendency to co-occur in the clinic.

## 2. Activation of the Wnt Pathway in Prostate Cancer

Multiple studies have reported a strong correlation between elevated Wnt signaling and prostate cancer (reviewed in [28,30]). Activation may occur at the level of the Wnt receptors/co-receptors, extracellular regulators, and intracellular components, either alone or in combination. Here we review the common drivers of Wnt activity, including known genetic drivers as well as several post-transcriptional and post-translational events that may elevate Wnt signaling to promote prostate cancer.

### 2.1. Deregulation of Intracellular Components 

#### 2.1.1. β-Catenin Gain of Function

**Figure 2:**
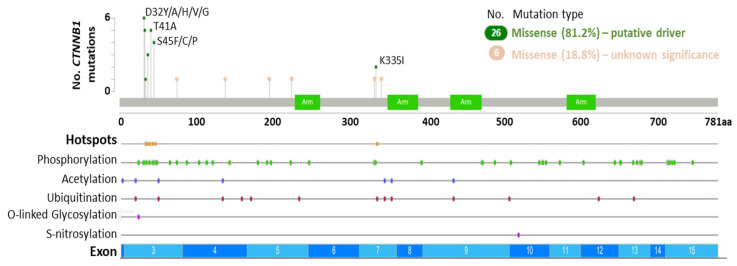
*CTNNB1* somatic mutations in prostate cancer. Diagram indicates *CTNNB1* mutation frequency in relation to exons and post-translational modifications. Data sourced from The Cancer Genome Atlas (TCGA) Firehose Legacy (primary: *n* = 492) [66] and the SUC2/PFC International Dream Team (metastatic: *n* = 444) [64] prostate adenocarcinoma datasets using cBioPortal [68,69] (Table S5). Germline mutations: 0/32 (0.0%). Somatic mutations: 30/32 (93.75%). Unknown mutational status: 2/32 (6.25%). Arm: Armadillo repeat.

The proto-oncogene β-catenin is a key mediator of canonical Wnt signaling that regulates the transcription of Wnt target genes and interacts with additional signaling pathways (e.g., AR, MAPK, and PI3K cascades) and E-cadherin to coordinate cellular processes such as cell adhesion, proliferation, survival, and stemness during development, regeneration and tumor growth [19,50,70]. In the absence of Wnt pathway stimulation, the destruction complex negatively regulates β-catenin activity via phosphorylation and ubiquitination events that result in cytoplasmic degradation [28]. CK1α phosphorylates β-catenin at S45, which in turn permits GSK3β-mediated phosphorylation of residue T41, and successive phosphorylation of S37 and S33 to produce a binding site for β-transducin repeat containing E3 ubiquitin protein ligase (β-TrCP) that targets β-catenin for degradation [71,72,73]. In prostate cancer, activating mutations that render β-catenin constitutively active are present in primary and metastatic cases, albeit most prevalent in metastatic cases (Table 1 and Tables S1–S5). *CTNNB1* mutations are predominantly missense single nucleotide polymorphisms, and 81.2% of the *CTNNB1* mutations detected are predicted to be oncogenic (Figure 2, Table S5). Hotspot mutations in prostate cancer include phosphorylation sites within exon 3 (D32Y/A/H/V/G, G34E, S37A/C/P/Y, T41A, and S45F/C/P) and in exon 7 (K335I) (Figure 2, Table S5). Pathogenic variants at the phosphorylation sites stabilize β-catenin by preventing CK1α/GSK3β-mediated phosphorylation, thus reducing β-TrCP binding affinity and β-catenin degradation, and K335 mutation has been linked to reduced *APC* binding and increased β-catenin activity [71,72]. The functional consequence of *CTNNB1* mutations at other non-phosphorylation sites remains to be determined and could lead to the discovery of new Wnt pathway genetic driver mutations. 

Therapeutic resistance in prostate cancer has also been linked to *CTNNB1* mutation and Wnt pathway deregulation. For instance, primary prostate cancer patients harboring *CTNNB1* genetic alterations positively correlate with earlier relapse [18], and *CTNNB1* mutations occur at a higher frequency in plasma cell-free DNA (cfDNA) from CRPC patients that progressed on enzalutamide [74]. A mCRPC study has also identified that genes in the Wnt/β-pathway (including *CTNNB1*) are more frequently mutated in patients exhibiting primary abiraterone/prednisone resistance [19]. Additionally, analysis of β-catenin by immunohistochemistry (IHC) has revealed that elevated β-catenin expression is associated with prostate cancer progression and worse overall survival [20,75,76]. For instance, the number of nuclear β-catenin and LEF1 double-positive epithelial cells is reported to be increased in high-grade prostatic intraepithelial neoplasia (HG-PIN) and metastatic prostate cancer specimens relative to benign prostate tissue [70]. Taken together, these findings indicate that β-catenin gain of function plays an oncogenic role during prostate tumorigenesis, metastatic progression, and therapeutic resistance.

#### 2.1.2. *APC* Inactivation

The tumor suppressor *APC* is a key component of the β-catenin destruction complex that can interact directly with β-catenin in the nucleus to negatively regulate canonical Wnt signaling and control cell proliferation, survival, differentiation, and migration [77]. *APC* can also interact with the cytoskeleton to mediate cell motility [78], and *APC* inactivation has been shown to stimulate the formation of LRP6 signalosomes independently of Wnt ligands via clathrin-mediated endocytosis to activate Wnt signaling [79]. Thus, *APC* can inhibit Wnt signaling via distinct mechanisms that include suppressing Wnt receptor activation and reducing β-catenin accumulation in the cytoplasm.

**Figure 3:**
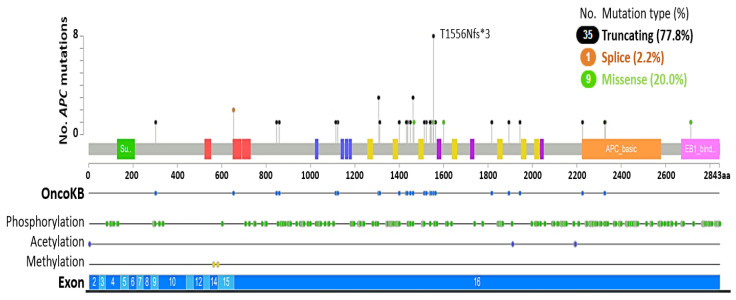
*APC* somatic and germline mutations in prostate cancer. Diagram indicates *APC* mutation frequency in relation to exons and post-translational modifications. OncoKB indicates alterations that are likely to be oncogenic (*n* = 36/45, 80%). Data sourced from The Cancer Genome Atlas (TCGA) Firehose Legacy (primary: *n* = 492) [66] and the SUC2/PFC International Dream Team (metastatic: *n* = 444) [64] prostate adenocarcinoma datasets using cBioPortal [68,69] (Table S6). Germline mutations: 2/45 (4.44%). Somatic mutations: 37/45 (82.22%). Unknown mutational status: 6/45 (13.33%).

*APC* was first identified through its association with familial adenomatous polyposis coli (FAP), a syndrome where inherited mutations that delete *APC* cause colorectal and intestinal adenomatous polyps, increasing the risk of developing cancer [80]. *APC* loss via somatic truncation mutation, deletion, and promoter hypermethylation is common in several human malignancies, including colorectal cancer (CRC), breast and non-small cell lung cancer (NSCLC) [75,76,81]. In prostate cancer, *APC* loss is also linked to homozygous deletion and truncation mutations in up to 7% of primary and 10.6% of metastatic cases (Table 1), resulting in the accumulation of β-catenin and elevated Wnt signaling. The majority of *APC* mutations are somatic, with truncating mutations (77.8%) such as T1556Nfs*3 being most common, while splice variants are uncommon (2.2%) (Figure 3, Table S6). A number of missense mutations (20%) have also been detected in *APC*, however the functional consequence of these alterations is unknown.

Genomic analysis of metastatic castration-sensitive prostate cancer specimens was also recently identified in 12% (50/424) of cases harboring an *APC* genetic alteration, predominantly truncation mutations, and homozygous deletion, although putative drivers including a splice mutation and structural variants were also evident [82]. Furthermore, *APC* loss has also been linked to abiraterone/enzalutamide treatment resistance in mCRPC [16], indicating aberrant activation of the Wnt pathway facilitates resistance to androgen/AR pathway directed therapies in the clinic.

#### 2.1.3. DVL Deregulation

DVL is a key cytoplasmic component of the Wnt cascade that transmits signals from upstream cell surface Wnt receptors/co-receptors by interacting with a multitude of cytoplasmic proteins to activate downstream effector cascades and coordinate canonical and non-canonical Wnt signaling events [83,84] (Figure 1). There are three mammalian DVL homologues (DVL1, DVL2, and *DVL3*) that are recruited to FZD receptors upon Wnt binding. Once bound to FZD receptors, DVLs are then phosphorylated and can form polymers that promote the formation of Wnt-FZD-LRP signalosomes [83]. These signalosomes can suppress the β-catenin destruction complex by recruiting AXIN/GSK3β to the plasma membrane, thus activating the Wnt/β-catenin pathway [83]. DVL can also interact with β-catenin in the nucleus to regulate the transcriptional activity of Wnt target genes [85]. In prostate cancer, *DVL1/2/3* are rarely mutated (<1%), however *DVL3* is amplified in up to 2.2% of primary cases, and as many as 8.8% of metastatic cases (Table 1 and Tables S1–S4). While the functional significance of *DVL3* amplification is currently unclear, *DVL3* has been found to participate in an adaptor complex that links insulin-like growth factor 1 receptor (IGF1R) to RAS, and depletion of *DVL3* (but not DVL1 or DVL2) can sensitize DU-145 CRPC cells to the IGF1R inhibitor AZ12253801 [86].

Interestingly, *DVL1* amplification and over-expression were observed in breast and prostate cancers respectively [87,88], while *DVL3* mRNA was significantly increased in pleural effusions from patients with lung cancer [89] and was over-expressed in NSCLC [90,91]. These findings suggest DVL may facilitate tumor growth and/or metastatic progression. Moreover, DVL1 and *DVL3* expression is increased in metastatic tumors compared to primary NSCLC, and DVL1 expression positively correlates with β-catenin expression metastatic NSCLC [91]. In support, *DVL3* mRNA and protein expression is elevated in metastatic oesophageal squamous carcinoma, and *DVL3* knockdown is reported to reduce oesophageal squamous carcinoma cell proliferation, invasion, and survival in vitro, while enforced *DVL3* expression can increase tumor growth in an oesophageal squamous cell carcinoma xenograft model [92]. Conversely, analysis of *DVL3* by IHC has indicated *DVL3* expression does not correlate with tumor stage, grade or survival in prostate and breast cancers [86], and homozygous deletion of *DVL1* and *DVL2* has also been observed in primary and metastatic prostate cancer (up to 6.8% and 4.3% incidence respectively, Tables S1–S4), and these events appear to be mutually exclusive (Figures S1–S4). Thus, further work to better understand the predictive value of DVLs and their mode of action in prostate cancer is needed. 

#### 2.1.4. Upregulation of Wnt Pathway Transcription Factors

Genetic analysis of large prostate cancer datasets has revealed that several co-activators of β-catenin-mediated transcriptional activity are amplified in prostate cancer, such as *BCL9* and *PYGO2* (Table 1) [64,65,66]. The transcription factor *BCL9* is over-expressed in a number of human malignancies, including prostate cancer, to promote tumor growth by upregulating the transcription of Wnt target genes [93]. Accordingly, the β-catenin:*BCL9* complex and its regulators present valuable anti-cancer therapeutic targets. For instance, large tumor suppressor kinase 2 (LATS2) has been shown to inhibit β-catenin:*BCL9* mediated transcription independently from its role in the Hippo pathway where it phosphorylates YAP, and the microtubule inhibitor nocodazole can induce LATS2 to suppress β-catenin:*BCL9* mediated transcription in human colorectal cancer cell lines [94]. *BCL9* is also a direct target of miR-30c, which negatively regulates *BCL9* to reduce Wnt target gene transcription. Moreover, miR-30c and *BCL9* expression inversely correlate with prostate cancer [93]. Recently, β-catenin-independent *BCL9* functions have also emerged through interactions with paraspeckle proteins in colorectal cancer, indicating *BCL9* oncogenic functions expand beyond the Wnt cascade [95].

*PYGO2* has recently been identified in functional genomics in vivo screens as an oncogenic driver of prostate cancer [96]. The β-catenin transcriptional co-activator *PYGO2* binds to methylated histone H3, lysine 4 (H3K4me) to activate Wnt/β-catenin dependent gene expression. *PYGO2* upregulation is associated with higher Gleason score and metastasis to lymph nodes and bone, and *PYGO2* overexpression increased in vivo tumor growth and lymph node invasion of immortalized LHMK prostate cancer cells (derived from primary prostate cells transformed with SV40 Large T, hTERT, Myc and PI3K) while *PYGO2* shRNA-mediated depletion reduced primary tumor burden and metastasis in the PC-3 xenograft model [96]. These findings strongly suggest *PYGO2* plays an oncogenic role in prostate cancer. Interestingly, high *PYGO2* expression has also been shown to positively correlate with earlier PSA biochemical recurrence, indicating *PYGO2* may also prove to be a valuable predictive biomarker for prostate cancer [97]. 

### 2.2. Wnt Receptor/Co-Receptor Deregulation

#### 2.2.1. FZD Receptors

Genetic aberrations in the seven-transmembrane family of Frizzled Wnt receptors (*FZD1-10*) are reported in a number of human solid cancers [98], including prostate cancer (Table 1 and Tables S1–S4) [64,66]. In primary and metastatic prostate cancer, FZD Wnt receptor genetic mutations are relatively uncommon (<0.6% and 0.23–2.1%, respectively, Tables S1–S4), and although several *FZD1-10* mutations have been shown to correlate with copy number alterations [64,65,66], it is not currently known if they alter Wnt receptor activity. Interestingly, *FZD6* and *FZD9* genes are commonly amplified in metastatic prostate cancer (10.5–23.2% and 3–5.6%, respectively), with reduced frequency observed in primary cases (3.1–6.3% and 0.8–1.3%, respectively) (Table 1). The frequency of *FZD1/2/3/4/5/7/8/10* gene amplification in primary prostate cancer is also relatively low (≤3%), with incidence increasing slightly in metastatic disease (≤4.5%) (Tables S1–S4). In addition, *FZD2*, *FZD4*, and *FZD8* mRNA upregulation is also reported in human metastatic prostate cancer cell lines [99], and *FZD2*, *FZD7*, and *FZD8* mRNA levels are increased in hormone depleted LNCaP cells [99]. Increased transcription of *FZD3/5/7/8* has also been linked to TMPRSS2-ERG (Transmembrane serine protease 2–ETS Transcription Factor ERG fusion protein) gene-fusion positive prostate tumors, and the ERG-regulator SOX9 has been shown to positively correlate with Wnt activity and the upregulation of Wnt pathway components, including *FZD7* mRNA in advanced prostate cancer models and primary prostate cancer clinical specimens [100,101,102,103,104]. Conversely, *FZD3* homozygous deletion is a common event both in primary and metastatic prostate cancer (1.5–12% and 2–10% of cases respectively, Table 1). These findings highlight the diverse nature of FZD receptor genetic variants in prostate cancer, which could influence both canonical and non-canonical Wnt signaling activity, and emphasize the need to better understand how they contribute to tumor growth. 

While functional analysis of FZD receptors in solid cancers is currently limited, it is reasoned that amplification or upregulation of FZD Wnt receptors could activate the Wnt cascade, whereas *FZD3* deletion could result in diminished Wnt signaling. However, compounding factors, including the expression of each FZD receptor and the activity status of all Wnt pathway components, are highly likely to impact the functional consequence of FZD copy number alterations. Interestingly *FZD8* is upregulated in advanced prostate cancer compared to benign prostate tissue, and *FZD8* knockdown in DU-145 and PC3-M cells significantly reduces cell migration and invasion, associated with downregulation of mesenchymal markers seen in both in vitro cell line studies and cell line-derived organoids [99], suggesting FZD8 mediates prostate cancer dissemination and epithelial-to-mesenchymal transition (EMT). Furthermore, *FZD8* knockdown in a chick chorioallantoic membrane (CAM) model of prostate cancer reduced tumor burden correlating with a reduction in vimentin expression [99]. Taken together, these data strongly suggest FZD Wnt receptors are amplified to increase Wnt signaling and promote prostate tumor growth and invasion. However, further research is required to identify the biological function of specific Wnt receptors during prostate cancer formation and progression. 

#### 2.2.2. LGR Deregulation

Leucine-rich repeat-containing G-protein coupled receptors (GPCRs) *LGR4*/*5*/*6* play a pivotal role in Wnt signaling and are well-characterized markers of stem cells that mediate stem cell activity in multiple tissues [105]. For instance, LGR5 is a marker of adult stem cells in the gastrointestinal tract and the bulge region of hair follicles, and *LGR6* is a marker of stem cells in the sebaceous gland and associated interfollicular epidermis in adult skin [105]. LGRs bind to the extracellular ligand R-spondin (RSPO1-4) and forms a complex with co-receptors RNF43/ZNRF3 to alleviate RNF43/ZNRF3-negative regulation of FZD receptors, permitting FZD receptors to transduce Wnt-signals intracellularly to activate downstream Wnt effector cascades [106,107,108] (Figure 1), and have been shown to be deregulated in several solid cancers [109,110,111,112]. 

In the prostate, LGR4 is essential for prostate development and stem cell differentiation, owing to LGR4-mediated regulation of Wnt, Notch, and Sonic Hedgehog signaling [113]. High levels of *LGR4* mRNA in prostate cancer also positively correlate with shorter recurrence free-survival in the clinic, and *LGR4* loss in the TRAMP mouse model of neuroendocrine prostate cancer delays the onset of prostate intraepithelial neoplasia (PIN) in vivo, associated with increased survival and reduced lung metastasis [114]. Moreover, *LGR4* knockdown in DU-145 mCRPC cells reduces the transcription of EMT and Wnt target genes, and decreases tumor burden in xenografts, suggesting LGR4 plays an oncogenic role in the prostate by potentiating Wnt signaling and EMT [114]. LGR5-positive cells are also reported to present a rare population of progenitor cells in regressed prostate epithelium post-castration in adult mice, and their presence is required for regeneration [115]. Little research has focused on *LGR6* in prostate cancer, although a previous study has shown *LGR6* mRNA is not expressed in primary luminal, basal, or stromal cells that were isolated by fluorescence-activated cell sorting (FACS) from adult mouse prostate tissue [116] and *LGR6* upregulation in ovarian cancer predict for poor prognosis [112]. In support of the notion that *LGR6* is oncogenic, *LGR6* shRNA-mediated knockdown in ovarian cancer cells is reported to attenuate stemness by inhibiting the Wnt/β-catenin pathway, and in vivo experiments have shown *LGR6* knockdown sensitizes the SK-OV-3 ovarian cancer xenograft model to chemotherapy (cisplatin) [112]. Collectively, these findings indicate *LGR4*/*5*/*6* upregulation increases Wnt signaling to facilitate tumor growth and therapeutic resistance in human cancers. However, two independent studies have also identified tumor-suppressive functions of *LGR6*, suggesting *LGR6* oncogenic and tumor-suppressive functions are likely to be tumor and tissue-type dependent [108,117]. 

*LGR4*/*5*/*6* are rarely mutated or amplified in primary prostate cancer (≤0.41% and ≤1.32% respectively, Table 1 and Tables S1–S4), suggesting that activation of the Wnt cascade in primary prostate cancer is not commonly bolstered by *LGR4*/*5*/*6* genetic aberrations. In metastatic prostate cancer, the mutation frequency remains low, with *LGR6* mutation being slightly more prevalent (1.4–1.8%, Table 1). However, the pathogenic nature of these variants remains to be determined. Conversely, *LGR4*/*5*/*6* high level gene amplification is more frequent, particularly for *LGR6* (*LGR4*: 2.1–2.5%, *LGR5*: 0–5%, *LGR6*: 5.7–7.4% incidence, Table 1 and Tables S1–S4) [64,66]. Accordingly, future work to establish if *LGR4*/*5*/*6* amplification in metastatic prostate cancer promotes Wnt signaling to facilitate advanced prostate cancer progression is needed.

#### 2.2.3. LRP Deregulation

The single-spanning transmembrane low-density lipoprotein receptor family members *LRP5* and *LRP6* are Wnt co-activators that are instrumental for Wnt signaling activity. Wnt-stimulated FZD1-10 and *LRP5*/*6* receptor complexes recruit DVL polymers to the plasma membrane, which in turn recruit AXIN/GSK3β to form signalosomes that inhibit the β-catenin destruction complex, enabling Wnt target gene expression (Figure 1). Human epithelial cancers frequently show *LRP5*/*6* over-expression, correlating with increased Wnt/β-catenin signaling, highlighting their oncogenic role [118]. Increased LRP6 phosphorylation has also been shown to correlate with poor prognosis in CRC [119]. *LRP5* and *LRP6* amplification is observed in up to 7.2% and 2.7% of metastatic prostate cancers, yet are relatively infrequent in primary prostate cancer (0–2.1%) (Table 1 and Tables S1–S4). It is tempting to speculate *LGR5*/*6* amplification increases Wnt signaling to promote prostate cancer progression, although functional experiments to address this are yet to be published. In support, *LRP5* knockdown in PC-3 cells reduced tumor burden and skeletal metastasis in xenografts [120], and small molecule inhibitors that reduce LRP6 expression and phosphorylation (e.g., niclosamide, salinomycin, and silibinin) can inhibit prostate cancer cell growth and increase apoptosis (reviewed in [118]). *LRP5* has also been shown to mediate the prostate cancer-induced formation of new bone in an ex vivo bone formation assay, indicating *LRP5* may contribute to the formation of prostate cancer skeletal metastases [121]. Furthermore, effective anti-cancer strategies involving *LRP5*-directed therapies remains a current conundrum, as Wnt signaling orchestrates normal bone formation and *LRP5* expression in osteoclasts results in tumor-suppression [122]. Accordingly, *LRP5*/*6* genetic variants are strongly associated with bone diseases, including osteoporosis and osteoarthritis [123].

*LRP5*/*6* are infrequently mutated in primary (≤0.61%) and metastatic (≤2.1%) prostate cancer, and are predominantly missense variants (Table 1 and Tables S1–S4) [64,65,66]. Although it is not yet clear how these genetic alterations affect tumor formation and progression, in other human cancers, some variants have been associated with reduced cancer risk, while others may increase this risk. For instance, the *LRP6* rs10743980 variant is linked to decreased risk of bladder cancer [124], whereas *LRP6* rs141458215 (p.T867A), p.N789S and p.W239L are associated with increased risk of colorectal cancer [125], reviewed in [118]. Of note, *LRP6* homozygous deletion was detected in up to 4.9% and 4.3% of primary and metastatic cases, respectively (Table 1 and Tables S1–S4) [64,65,66]. Together, these findings indicate additional work is needed to establish how *LRP5*/*6* genetic alterations contribute to prostate cancer growth, to gain new insight into how these Wnt co-receptors function, and to discover new therapeutic opportunities to treat this lethal disease.

#### 2.2.4. RYK Upregulation

RYK is a member of the receptor tyrosine kinase family that serves as a Wnt co-receptor, binding Wnt ligands to activate the canonical and non-canonical Wnt pathways [28,126]. To date, RYK is generally considered to be a pseudokinase as catalytic activity remains to be identified, and perhaps the best characterized RYK-Wnt interaction is with the non-canonical Wnt ligand *Wnt5a* [28,126]. RYK expression has been linked to several human cancers, including glioma, ovarian, gastric, and prostate malignancies [103,127,128,129]. *RYK* mRNA and nuclear RYK protein expression positively correlated with Gleason score, yet increased RYK expression alone does not appear to predict poor prostate cancer survival [103,130]. Knockdown of *RYK* in PC-3 prostate cancer cells reduces *Wnt5a*-induced apoptosis in vitro without altering proliferation via an unclear mechanism. *RYK* knockdown in glioma cells suppressed matrix metalloproteinase-2 (MMP2) and *Wnt5a*-induced invasion [127], suggesting RYK can regulate extracellular matrix degradation and tumor invasive capacity. 

Analysis of clinical prostate cancer genomic datasets indicates that *RYK* gene amplification occurs in 2–2.7% and 2.7–7.2% in primary and metastatic cases, respectively (Table 1 and Tables S1–S4) [64,65,66], indicating a higher frequency occurs in advanced disease. Whether RYK amplification is a pathogenic variant in prostate cancer is currently unknown, however, RYK over-expression in mouse fibroblasts induces anchorage-independent growth in vitro and increases tumorgenicity in vivo [129], supporting an oncogenic role. Interestingly, RYK upregulation was also reported in DU-145 prostate cancer cells in response to the anti-estrogen ICI 182,780, reflecting ER-β/Nf-κB signaling cross-talk and the presence of a cis-acting NF-κB binding element in the promoter region of RYK [130]. Hence, while current findings point towards an oncogenic role for RYK in several epithelial cancers, including prostate cancer, a deeper understanding of RYK functions during normal prostate tissue homeostasis and prostate tumorigenesis is needed to ascertain the mechanism(s) whereby this receptor tyrosine kinase could contribute to prostate cancer growth. 

#### 2.2.5. RNF43 and ZNRF3 Deregulation

The cell-surface transmembrane Wnt co-receptors RNF43 and ZNRF3 are homologous RING-domain containing E3 ubiquitin ligases that negatively regulate the Wnt pathway by targeting FZDs, and Wnt co-receptors (e.g., *LRP5*/*6*) for ubiquitin-mediated degradation [131,132] (Figure 1). Consequentially, the inactivation of RNF43 and ZNRF3 promotes Wnt signaling by stabilizing FZD receptors and Wnt co-receptors at the plasma membrane. *RNF43* and *ZNRF3* are also Wnt target genes, thus providing a negative feedback loop whereby activation of the Wnt pathway results in *RNF43/ZNRF3* gene transcription to switch off the pathway [131,132]. In addition, RNF43 is also reported to sequester TCF4 to the nuclear membrane in the context of β-catenin hyperactivation [133] and can bind DVL to suppress non-canonical signaling [134]. Collectively, these findings indicate the canonical and non-canonical Wnt pathways are attenuated by RNF43, both at the receptor level and transcriptionally.

RNF43 and ZNRF3 are regarded as tumor suppressors in multiple cancer types, such as gastric, ovarian, pancreatic, endometrial, and colorectal cancers [135,136,137,138,139]. Several *RNF43/ZNRF3* genetic variants have been detected in human malignancies, however recent work in colorectal cancer has highlighted the need to determine which variants are pathogenic [135,136,137,138,139]. In prostate cancer, *RNF43* genetic mutations are present in primary and metastatic disease (≤1% and 1.8–2.5%, respectively, Tables S1–S4) [64,65,66], with around half of the variants occurring in the C-terminus. Indeed, 7/15 variants detected in the metastatic prostate cancer SUC2/PCF IDT dataset were in the C-terminus [64], including 5 cases with the G659Vfs*41 variant, which activates Wnt signaling in colon cancer [139]. 

In regard to *ZNRF3*, 1.8% of metastatic prostate cancer patients are reported to harbor genetic alterations, in comparison to just 0.2–0.44% of patients with the primary disease (Tables S1–S4) [64,65,66]. Interestingly, *ZNRF3* deep deletions are also observed in primary and metastatic prostate cancer (≤2%) (Tables S1–S4), potentially promoting Wnt signaling via the stabilization of FZD receptors and Wnt co-receptors at the plasma membrane. Nevertheless, the frequency of *ZNRF3* genomic loss was recently reported to be markedly higher (29.9% of metastatic and 9.54% of localized prostate cancers), and ZNRF3 loss positively correlates with biochemical recurrence and metastatic relapse of localized disease [140]. Future investigations exploring the role of clinically relevant genetic aberrations in *RNF43* and *ZNRF3* during primary and metastatic prostate cancer will be important to ascertain their impact on Wnt signaling. For example, mutations to *RNF43/ZNRF3* could deregulate the turnover of FZD Wnt receptors on the cell surface resulting in cells becoming hypersensitive to Wnt ligands, which could be missed when analyzing mutational and transcriptomics data. In addition, a deeper understanding of how these receptors are transcriptionally/post-translationally regulated in prostate cancer is also warranted. For instance, recent work has uncovered RNF43 phosphorylation is required for RNF43 to negatively regulate Wnt signaling, and deregulation of RNF43 phosphorylation can increase oncogenic activity [141].

### 2.3. Deregulation of Extracellular Wnt Pathway Regulators

#### 2.3.1. Wnt Ligands 

There are 19 Wnt ligands that bind with varying affinities and specificities to the FZD receptors to activate Wnt signaling. In prostate cancer, a number of Wnt ligands were found to be upregulated in patient tumors and/or circulating tumor cells (CTCs), including non-canonical Wnt ligands (*Wnt5a*, Wnt7b, and Wnt11) and Wnt/β-catenin ligands (Wnt16), reviewed in [28]. Activation of non-canonical Wnt signaling via *Wnt5a* has been linked to anti-androgen resistance, disease progression, and metastasis [19,20,109]. For instance, *Wnt5a*-FZD2 non-canonical Wnt signaling that activates the FYN-STAT3 signaling axis to promote EMT is reported to positively correlate with higher Gleason score and increased expression of EMT markers, furthermore a novel gene signature incorporating non-canoncial Wnt pathway and EMT genes can predict for biochemical recurrence [101]. *Wnt5a* from the osteoblastic niche has also been shown to induce prostate cancer cell dormancy in bone in the intra-tibial PC-3 xenograft model, involving the inhibition of Wnt/β-catenin signaling through the potentiation of ROR2-SIAH2 signaling [142]. In addition, *Wnt5a* expression in localized prostate cancer has been shown to correlate with a favorable outcome [143]. These data indicate that Wnt ligands play divergent roles dependent on several parameters, including receptor availability, tissue/cell type, and the stage of tumorigenesis. 

The observed increase in Wnt ligand expression in prostate cancer may augment Wnt signaling and can occur via multiple mechanisms, including AR- or FOXB2-mediated transcriptional regulation of Wnt genes [67,144,145], as well as post-translational events such as N-glycosylation and acylation via palmitoylation that are required for Wnt ligand folding and/or secretion (reviewed in [146]). Genetic mutations in Wnt genes are generally infrequent in primary and metastatic prostate cancer (0–1% and 0–1.8% incidence respectively, Tables S1–S4, pathogenicity unknown), while gene amplification is slightly more prevalent (Primary: 0–2.8%, Metastatic: 0–3.8%, Tables S1–S4), particularly *WNT2* and *WNT16* (Tables S1–S4) [64,65,66].

#### 2.3.2. DKK

The Dickkopf family is comprised of 5 secretory proteins, DKK1–4 and SOGGY. DKK1/2 antagonizes the Wnt/β-catenin pathway through extracellular interactions with the Wnt co-receptors *LRP5*/*6*, which prevents the formation of FZD:LRP complexes (Figure 1). DKK1/2 Wnt/β-catenin signaling suppression is potentiated by interaction with the co-receptor KREMEN (KREMEN1/2), whereas *DKK4* interactions with *LRP5*/*6* and KREMEN are less well characterized [147]. The role of DKK3 and Soggy in Wnt/β-catenin signaling is also unclear, as they are not known to interact with *LRP5*/*6* or KREMEN [147,148]. DKKs can also regulate the Wnt/PCP pathway [149,150], adding to the complexity of Wnt signaling regulation [147,148]. DKK-mediated cellular processes can also occur independently of the Wnt cascade. For instance, DKK1 interactions with the receptor cytoskeleton-associated protein 4 (CKAP4) can mediate proliferation in a Wnt independent manner [144]. Loss of *APC* in intestinal cancer cells or loss of PTEN in melanoma cells has also been shown to induce DKK2 expression, which coordinates tumor immune evasion via *LRP5* and suppression of STAT5 signaling [145]. 

Currently, DKKs are considered to play a dual role in cancer, possessing both oncogenic and tumor-suppressive functions depending on the context and tumor type (reviewed in [147]). In prostate cancer, *DKK1–4* genetic mutations are rare in primary and metastatic prostate cancer patients (0–0.68% incidence, Tables S1–S4) [64,65,66]. In comparison, *DKK1* gene amplification and homozygous deletion are slightly more frequent (primary: 0–0.9% and 0.9–1.8% respectively, metastatic: 3.3–4.5% and 1.2–1.6% respectively, Tables S1–S4) [64,65,66], with *DKK1* amplification being most prevalent in metastatic cases. DKK1 protein overexpression has been observed in serum and advanced prostate cancer tissue specimens and is associated with poor prognosis, increased tumor growth, immune evasion, and bone metastasis [103,151,152,153,154]. Conversely, *DKK1* homozygous deletion in prostate cancer is predicted to increase Wnt/β-catenin signaling via stabilization of FZD/LRP receptors at the plasma membrane. However, this remains to be determined and is likely to be dependent on the expression/function of other Wnt pathway components. 

DKK2 can both activate and inhibit Wnt/β-catenin signaling, depending on the cellular context, such as *LRP5*/*6* abundance and DKK1 expression, as DKK1 can inhibit DKK2 [147,148]. In human prostate cancer cells, DKK2 depletion is reported to suppress cell proliferation and invasion, attributable to reduced β-catenin and Wnt transcriptional targets CyclinD1 and Myc [155]. DKK2 is also reported to be upregulated in prostate cancer, however, the sample size and tumor stage assessed are unclear [155]. *DKK2* gene amplification and homozygous deletion are rare in prostate cancer, with gene amplification being slightly more frequent in metastatic cases (primary: 0–0.7% and 0.3–0.4% respectively, metastatic: 1.4–1.8% and 0–0.7% respectively, Tables S1–S4) [64,65,66]. 

Similarly, *DKK3* (also known as REIC, reduced expression in immortalized cells) gene amplification and homozygous deletion are also infrequent events in prostate cancer (0.2–1.5% and 0.2–0.6% incidence, respectively) (Tables S1–S4) [64,65,66]. DKK3 protein expression in prostate tumors is generally down-regulated owing to promoter methylation, whereas DKK3 is highly expressed in the surrounding stroma and endothelium [156]. Previous work has indicated DKK3 loss in prostate cancer cells can activate TGF-β signaling to facilitate tumor progression [157] whilst inducing DKK3 expression in the surrounding stroma to suppress tumor growth [158], emphasizing the importance of DKK3 in regulating tumor-stroma interactions during prostate tumorigenesis. Notably, doxycycline-inducible expression of DKK3 in LNCaP prostate cancer cells reduced cell proliferation but did not alter β-catenin cytoplasmic levels or inhibit Wnt/β-catenin signaling, consistent with the fact that DKK3 does not bind to the Wnt co-receptors *LRP5*/*6* or KREMEN [159]. DKK3 overexpression has also been linked to increased apoptosis in human prostate cancer cells involving JNK activation, indicating DKK3 may regulate non-canonical Wnt signaling in this setting [160].

*DKK4* appears to play a similar role to DKK1 to antagonize the Wnt/β-catenin pathway, and relative to all other DKK family members, genetic variants in *DKK4* are more common in prostate cancer (Table 1 and Tables S1–S4) [64,65,66]. *DKK4* gene amplification occurs in as many as 6% of metastatic prostate cancers, while homozygous deletion has also been observed in both primary (4–5.5%) and metastatic cases (1.6–3.6%) (Table 1). Although poorly studied in prostate cancer, both downregulation and upregulation of *DKK4* are reported to promote tumor progression in other human epithelial malignancies through the activation of Wnt/β-catenin signaling or other oncogenic cascades (e.g., non-canonical Wnt/JNK signaling or the MAPK cascade), respectively (reviewed in [161]). Interestingly, the PROMOTE study exploring genomic profiles from mCRPC patients before treatment with abiraterone acetate/prednisone revealed that *DKK4* loss and Wnt pathway activation correlate with treatment resistance [19] indicating a role in drug resistance. 

#### 2.3.3. RSPO

The RSPO family of secreted proteins is comprised of four members, RSPO1–4, which stimulate the Wnt pathway extracellularly by binding to Wnt co-receptors such as *LRP5*/*6*, *LGR4*/*5*/*6*, RNF43, and ZNRF3 [162]. RSPOs promote FZD receptor stabilization by inducing the ubiquitylation and degradation of RNF43 and ZNRF3 [163]. Although the mode of action of RSPOs remains to be fully established, they are considered to activate both canonical and non-canonical Wnt signaling pathways through their interactions with various Wnt co-receptors [162]. They can promote growth and luminal differentiation in prostate organoids [164]. The oncogenic function of RSPOs has been well documented, with a gain of function translocations and gene-fusions reported to activate WNT signaling by increasing the abundance of FZD-LRP complexes at the plasma membrane and sensitizing cells to Wnt ligands [165]. *RSPO1-4* genetic mutations are infrequent in primary and metastatic prostate cancer (0–0.6% incidence, Tables S1–S4 [64,65,66]), however, *RSPO2* gene fusions that increase *RSPO2* expression have previously been identified in 1.3% of mCRPC patients [67], and *RSPO2* genetic variants are common in prostate cancer, especially in metastatic disease (Table 1). 

*RSPO2* gene amplification occurs in up to 6.5% and 21% of primary and advanced prostate cancers, respectively, and has a strong tendency to co-occur with *FZD6* overexpression (Table 1, Figures S1–S4, [64,65,66]). Thus, *RSPO2* upregulation in prostate cancer provides a direct mechanism for elevated Wnt signaling that can promote tumor growth. In contrast, gene amplification of either *RSPO1*, *RSPO3*, or *RSPO4* is low (primary: <0.7%, metastatic: <2%, Tables S1–S4 [64,65,66]). However, the transcriptional analysis revealed that low levels of *RSPO3* mRNA are a prognostic marker for poor biochemical relapse-free survival [166]. Of note, upregulation of *RSPO3* mRNA was also observed in prostate cancer stroma [167], thus the role of RSPO3 during prostate cancer is unclear, with evidence pointing towards cell-type-specific functions. *RSPO3* siRNA-mediated knockdown in human prostate cancer cell lines increases invasive capacity in vitro, supporting a tumor-suppressive role for RSPO3 in prostate cancer [166], whereas tissue regeneration upon androgen replacement post-androgen deprivation involves the upregulation of RSPO3 by mesenchymal cells [168]. Collectively, these findings indicate the potential predictive value of combining genomic and transcriptomic data analysis to determine the degree of R-spondin upregulation in prostate cancer and the requirement for further work to explore if R-spondin signaling coordinates tumor-stroma interactions to facilitate tumorigenesis and/or therapeutic resistance.

#### 2.3.4. sFRPs

The sFRP family contains five glycoproteins, *SFRP1*, SFRP2, SFRP3 (FRZB), SFRP4, and SFRP5, which are generally regarded as tumor suppressors owing to their ability to negatively regulate the Wnt pathway [169]. sFRPs can be excreted by exosomes to suppress the Wnt pathway and prevent tumor growth via multiple mechanisms. For instance, sFRPs can directly bind and sequester Wnt ligands to prevent Wnt:FZD interactions or form a non-operational complex with FZD receptors. More recently, all sFRPs have been shown to interact with β-catenin via their N-terminus in the nucleus to suppress β-catenin transcriptional activity by modulating TCF4 recruitment [169], however it is still not clear how sFRPs enter the nucleus. Interestingly, the C-terminus of SFRP3 and SFRP4 can also interact with the C-terminus of β-catenin to promote transcription of Wnt/β-catenin target genes when β-catenin levels are high or SFRP3/4 levels are low in the nucleus, indicating divergent roles [169]. Controversially, sFRPs have also been shown to possess oncogenic functions [170], although the mechanisms underpinning this remain to be fully elucidated [171,172,173]. 

SFRPs are frequently lost in human cancers, including prostate cancer, predominantly owing to epigenetic hypermethylation/inactivation or miRNA transcriptional silencing. A large prostate cancer tissue microarray study identified granular cytoplasmic SFRP4 over-expression positively correlated with aggressive disease, early PSA-recurrence, and genomic instability in ERG negative prostate cancers [174]. A smaller study has also reported membranous expression of SFRP4 predicts a favorable prognosis in localized prostate cancer using a non-commercial antibody [175]. *SFRP1* expression inversely correlates with β-catenin in prostate cancer, where low expression of *SFRP1* predicts a worse outcome, and *SFRP1* downregulation has been linked to epigenetic inactivation [176,177]. In androgen-sensitive prostate cancer cells, *SFRP1* has been shown to inhibit AR transcriptional activity independently of Wnt/β-catenin signaling [171]. *SFRP2* and *SFRP5* promoters are also frequently hypermethylated in primary prostate cancer (65% and 60%, respectively), with prevalence significantly reduced in neoplastic and benign lesions [178]. *SFRP1* expression has also been shown to be regulated by microRNAs in prostate cancer, such as miR1301-3p and miR1260b [172,173]. While genetic mutations in genes encoding sFRPs are uncommon in primary and metastatic prostate cancer (<0.6%, Tables S1–S4), gene amplification, and homozygous deletion are generally more frequent, particularly in *SFRP1* (Table 1 and Tables S1–S4). 

#### 2.3.5. WIF1

Wnt inhibitory factor 1 (WIF1) is a negative regulator of canonical Wnt/β-catenin signaling that binds to secreted Wnt ligands, preventing Wnt interaction with their cognate receptors. Genetic variants in WIF1 are rare in prostate cancer (Tables S1–S4). However, *WIF1* epigenetic changes such as promoter methylation have been identified in several human malignancies (including prostate cancer), resulting in WIF1 depletion and augmented Wnt signaling [28]. *WIF1* downregulation has also been linked to abiraterone acetate/prednisone resistance, having been observed in mCRPC patient biopsies that did not respond to treatment [19]. Moreover, several human prostate cancer cell lines (LNCaP, LAPC4, PC-3, DU-145, C4-2B, PC3-M, and LN4, but not 22Rv1) lack *WIF1* mRNA expression owing to promoter hypermethylation [179]. Restoration of WIF1 in PC-3 cells induced a morphological change from a fibroblastic to an epithelial appearance, with a concomitant reduction in EMT markers such as N-cadherin and fibronectin [34]. These findings suggest WIF1 plays a tumor-suppressive role in prostate cancer, preventing EMT. In support, WIF1 overexpression significantly reduces the motility and invasiveness of PC-3 cells in vitro, and reduces tumor burden in PC-3 xenografts in vivo [34]. Tissue recombination experiments have also revealed WIF1 overexpression in the urogenital sinus (UGS) mesenchyme inhibits prostate development [180], indicating WIF1 is required for both normal prostate development and tissue homeostasis. 

## 3. Modelling Wnt-Driven Prostate Cancer 

### 3.1. Genetically Engineered Mouse Models (GEMMs) of Prostate Cancer

A broad array of GEMMs have been developed to study the functional consequence of deregulated Wnt pathway components in prostate cancer, including the conditional deletion of *Apc* or constitutive activation of β-catenin specifically within the murine prostate epithelium (summarized in Table 2). As such, GEMMs have played a significant role in furthering our molecular understanding of clinically relevant genetic alterations and prostate cancer biology. 

Inactivation of *Apc* driven by *Cre-LoxP* mediated excision of exon 14 causes prostate hyperplasia by 4.5 weeks of age and keratinized squamous metaplasia and adenocarcinoma by 7 months of age with high levels Wnt signaling detected by IHC analysis of nuclear β-catenin [181]. No metastatic progression was observed by 15 months of age [181]. Similarly, we and others have previously reported that targeting constitutive activation of β-catenin to the prostate epithelium using *Cre-LoxP* technology to excise *Ctnnb1* exon 3 (where GSK3β phosphorylation sites reside for ubiquitin-mediated degradation) causes PIN by 14 weeks [185,186,189,192]. PIN lesions featured the characteristic keratinized squamous metaplasia associated with *Apc* bi-allelic loss and progressed to locally invasive prostate carcinoma by 42 weeks with rare keratinized squamous metaplastic foci. Further aging has also revealed a low incidence of visceral metastasis [18]. Interestingly, a similar study did not observe progression to adenocarcinoma [184], probably owing to differences between the timepoints analyzed.

While *Probasin* (*PB*) promoter mediated Cre-recombinase expression driven by the *PBCre4* (*ARR2*) transgene (described in [201]) has been widely employed to explore β-catenin activation and *Apc* loss in both luminal and basal prostate epithelial cells, several experiments have also investigated luminal cell specific targeting using *Nkx3.1-Cre* or the inducible *Nkx3.1-Cre^ERT2^* construct that also drive *Nkx3.1* loss (Table 2). *Nkx3.1-Cre*-mediated activation of β-catenin results in neonatal lethality, however, ex vivo culture of embryonic prostate glands from these mice revealed β-catenin stabilization promotes the formation of squamous epithelia during prostate development in the presence or absence of DHT, indicating aberrant Wnt signaling induces trans-differentiation to a squamous cell fate in the prostate independently of androgen signaling [186]. In addition, canonical Wnt signaling and Nkx3.1 are reported to function in a positive feedback loop to regulate prostate bud growth and luminal epithelial differentiation in mice [202]. Wnt signaling occurs in the urogenital mesenchyme and prostate epithelial buds during mouse prostate development, and the addition of Wnt antagonists (DKK1-3) reduces prostate budding and inhibits Nkx3.1 expression in UGS cultures, as well as differentiation of luminal epithelial cells [202]. In the adult prostate, conditional expression of stabilized β-catenin in the stroma (*Col1a2Cre-ERT2 Ctnnbl^+/^*^Δ*ex3*^) causes prostate weight loss and reduced prostate epithelial cell proliferation, whereas loss of β-catenin in the stroma (*Col1a2Cre-^ERT2^ β-cat^fl/fl^*) increases prostate weight, indicating stromal Wnt/β-catenin activity may suppress prostate epithelial proliferation [200]. Homozygous deletion of *Ctnnb1* in mouse UGS has shown that β-catenin is essential for embryonic prostate development, whereas conditional deletion in adult prostate epithelial cells has revealed β-catenin is surprisingly dispensable for normal adult prostate tissue homeostasis, as well as hormone-sensitive and castration-resistant *Pten*-deficient prostate cancer growth [186,187].

Surgical castration of β-catenin-stabilized or *Apc*-depleted *PBCre4*-driven prostate cancer models at a timepoint when tumors are prevalent causes CRPC despite some initial partial sensitivity, indicating activation of the Wnt pathway promotes CRPC growth [181,184]. In support, castration resistance has also been observed in *Nkx3.1-Cre^ERT2^ Ctnnb1^+/^*^Δ*ex3*^ mice [188], although others have reported early castration in this model causes tumor regression [18]. These differences could reflect the disease stage at the time of castration, the period of time selected for histopathological analysis post-castration, and/or the genetic background. Of note, surgical castration of *Nkx3.1-Cre^ERT2^Apc^fl/fl^* mice with HG-PIN also causes disease regression [188]. 

Stabilization of β-catenin has also been shown to cooperate with an activating mutation in *KRas* (*KRas^G12V^*) to accelerate disease progression [185], and with *Pten* loss to induce metastatic disease (±*KRas^G12V^*) [18,186,189]. Analysis of triple mutants (*PBCre4 Ctnnb1^+/^*^Δ*ex3*^ *Pten^fl/fl^; K-Ras^+/G12V^)* revealed these three genetic alterations could synergize to drive aggressive tumor progression relative to double or single mutants via increased mTORC1 activity [189]. Deletion of the leucine zipper tumor suppressor 2 (*Lzts2*) that negatively regulates the Wnt pathway through β-catenin interactions has also been shown to synergize with *Pten* mono-allelic loss to induce early tumor onset and accelerate prostate cancer progression relative to single mutants [191]. Moreover, *LZTS2* and *PTEN* deletion frequently co-occur in human malignancies, including prostate cancer [191]. *Apc* loss has also been shown to synergize with MYC (M) overexpression and *Pten*-deletion (Pt) or p53 (P) loss to increase metastatic potential in *MPtApc* and *MPApc* electroporation (EPO) GEMMs respectively [198]. Gene set enrichment analysis (GSEA) of RNA sequencing data from *MP* EPO-GEMM tumors also revealed a Wnt/β-catenin signature, and *MP* EPO-GEMMs with high Wnt signaling showed a greater frequency of metastasis to the liver [198]. Micrometastases have also been observed in transgenic mice when *Apc* depletion occurs simultaneously with loss of the TGFβ type II receptor (*Tgfb2*), with a concomitant reduction in cell senescence [195]. *Apc* loss has also been shown to cooperate with hepsin overexpression or *Smad4* loss to promote invasive prostate cancer, further indicating that invasive progression in *Apc*-deficient prostate cancer requires an additional genetic alteration [196,197].

Notably, murine prostate tumors driven by β-catenin stabilization and *Pten* deletion show accelerated progression relative to single mutants and can also develop mCRPC following surgical castration, where metastatic growth is AR-deficient and β-catenin positive [18]. Interestingly, 43% of primary prostate adenocarcinomas and metastasis specimens with a *CTNNB1* mutation are reported to be *PTEN* deficient [18], highlighting the clinical relevance of these models. Moreover, ADT-treated patients with low PTEN and high β-catenin expression have significantly worse overall survival compared to those with high PTEN and low β-catenin [18]. Murine *Ctnnb1*-mutant prostate cancer spheroids are also reported to be enzalutamide resistant but responsive to *Wnt5a* loss [18]. Functional analysis indicated *Wnt5a* is induced by activated β-catenin to sustain nuclear Myc and NFkBp65 (RelA) and inhibit AR expression from facilitating the growth of mouse *Ctnnb1*-stabilised cells, further highlighting the complex relationship between Wnt and AR signaling [18]. Indeed, *PBCre4*-driven co-expression of a human AR transgene and stabilized β-catenin in mouse prostate epithelium accelerates tumorigenesis, increases invasive progression, and significantly reduces survival relative to β-catenin stabilization alone [55]. Aberrant expression of AR bearing short polyglutamine (polyQ) tracts (e.g., hAR12Q) and stabilized β-catenin has also been shown to induce the early onset of tumors and accelerate invasive prostate cancer progression relative to those carrying long polyQ tracts (e.g., hAR48Q) [193]. Short polyQ tracts have previously been linked to an increased risk of prostate cancer and aggressive disease, particularly in African American patients [193].

In support of the non-canonical Wnt ligand *Wnt5a* playing an oncogenic role in prostate cancer, *Wnt5a* depletion in the TRAMP mouse model of neuroendocrine prostate cancer harboring an *AR*^T877A^ mutation suppresses tumor onset and progression [199]. Moreover, *Wnt5a* is reported to reduce epithelial proliferation in adult wild-type proximal prostate ducts in mice [200]. Importantly, co-treating a model of β-catenin activated and *Pten*-deficient prostate cancer with ADT (surgical castration) and the porcupine inhibitor LGK974 that blocks the secretion of Wnt ligands reduced tumor burden and proliferation in vivo, indicating combining AR and Wnt pathway targeted therapies are efficacious against Wnt-driven prostate cancer [18]. 

Prostate cancer GEMMs have also identified that δ-catenin inactivation (which regulates E-cadherin stability and can suppress the Wnt/β-catenin pathway as a member of the β-catenin destruction complex), can promote rapid prostate tumor progression in the *PBCre4* Hi-Myc model [194]. Taken together, GEMM models have characterized several genetic drivers of the Wnt pathway, gained valuable insights into the synergistic relationship between the Wnt pathway and the RAS, PI3K/PTEN, MYC, and AR signaling, and strongly implicate the Wnt cascade in prostate cancer metastasis and androgen/AR-directed therapy resistance. Nevertheless, current research has primarily focused on assessing canonical Wnt/β-catenin pathway components. Future work exploring key components of non-canonical Wnt/PCP and Wnt/Ca2+ signaling during normal adult prostate tissue homeostasis and prostate cancer tumorigenesis is warranted.

### 3.2. Human Prostate Cancer Xenograft Models

A number of well-established human prostate cancer cell lines have been heavily employed in prostate cancer subcutaneous xenograft and experimental metastasis studies (reviewed in [203,204]). In addition, a wide variety of prostate cancer patient-derived xenograft (PDX) models have also been generated to model human prostate cancer [204,205,206]. PDX models recapitulate phenotypic and molecular characteristics of the donated patient tumors, including disease subtype, tumor heterogeneity, and genetic alterations, and are, therefore, a valuable resource for preclinical trials (albeit in an immunocompromised setting). For instance, the PNPCa PDX model derived from a treatment naïve soft tissue metastatic prostate cancer sample is reported to carry truncating mutations in *RNF43* and *APC*, while the bone-metastatic prostate cancer PDX model LAPC9 has mutations in both *APC* and *CTNNB1* (missense variation and in-frame insertion, respectively) [205]. The LuCAP models 167, 170.2, and 189.3 have also been shown to carry Wnt pathway genetic alterations [206]. LuCAP 167 has a somatic coding mutation in *LRP5*, together with a copy number gain in *RSPO2* that is also found in LuCAP 170.2, and LuCAP 189.3 carries somatic mutations in both *CTNNB1* and *WNT1* [206]. Thus, these models are clinically relevant preclinical tools to investigate the treatment of Wnt-driven prostate cancer.

### 3.3. Organoids

The development of 3D organoid cultures for both normal and tumor prostate epithelial cells has been instrumental for rapid functional genetic and preclinical drug screens in vitro, as organoids retain primary tissue heterogeneity, self-renewal capacity, and multi-lineage differentiation [170,207,208]. Recently, the effects of Wnt activity on mouse basal stem cell-derived prostate organoids were examined, revealing organoids derived from *Apc*-deficient mouse prostate basal cells were significantly larger and displayed complex branching relative to wild-type controls, indicating Wnt signaling promotes basal stem cell activities and organoid growth [209]. Furthermore, DHT treatment reduced Wnt target gene expression, suggesting androgen signaling negatively regulates Wnt signaling in this setting [209]. 

It is also possible to develop organoids from PDX models, known as PDX-Os. These include the LuCAP models 167, 170.2, and 189.3, which retain the Wnt pathway genetic alterations present in the PDX tumors (outlined above) [206]. Recently, a panel of patient-derived organoids (PDOs) was generated from both primary and metastatic prostate cancer specimens, and notably, the P20-11-Lg PDO line derived from a hormone naïve prostate cancer lung metastasis sample harbors an activating *CTNNB1* mutation (p-Ser45Pro) in addition to *TMPRSS2-ERG* fusion and *PTEN* genetic alterations (pThr319fs and c.209 + 2T > A), thus providing a valuable clinically relevant model [210]. PDOs have also been valuable in assessing differential treatment response, and the preclinical exploration of precision medicines. For instance, MC-PRX04 PDOs were derived from a CRPC patient responsive to abiraterone acetate/prednisone (AA/P) treatment, whereas MC-PRX01 and MC-PRX05 PDOs were derived from two separate patients that were both insensitive to AA/P treatment [19]. Interestingly, abiraterone resistance was overcome in MC-PRX01 and MC-PRX05 PDOs when combined with the tankyrase inhibitor XAV939 that inhibits Wnt signaling by blocking tankyrase 1 and 2 mediated Axin degradation, resulting in reduced pregnenolone-induced organoid growth relative to monotherapy. This contrasted with the MC-PRX04 AA/P-sensitive PDO model, where XAV939 treatment in combination with AA showed similar organoid growth suppression compared to AA alone [19]. Thus, organoids are emerging as an important preclinical tool for rapid drug screening, however, a broad range of models is required to encompass the plethora of oncogenic drivers in prostate cancer patients, and to continually recapitulate clinical disease as the treatment landscape evolves.

## 4. Wnt Signaling and Prostate Cancer Metastasis

**Figure 4:**
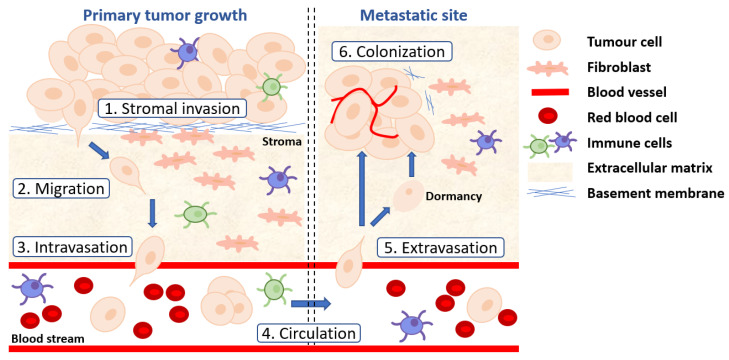
The metastatic cascade. (**1**) Tumor cells disseminate from the primary tumor site by invading through the basement membrane via proteolytic disruption of collagens and laminins. (**2**) Tumor cells adopt an EMT phenotype to migrate and invade through the surrounding stroma, breaking down the ECM. (**3**) Tumor cells enter the vasculature (intravasation) as single cells or as a cluster of cells. (**4**) CTCs survive in the circulation. (**5**) CTCs extravasate at distant sites through ruptured blood vessels. (**6**) Disseminated tumor cells colonize the metastatic site to form a secondary tumor by overcoming the harsh microenvironment, undergoing MET and establishing new vasculature. Alternatively, disseminated tumor cells enter a state of dormancy with potential to colonize.

Genomic profiling of metastatic prostate cancer (Table 1 and Tables S1–S4), functional genetic analysis of Wnt pathway genetic drivers in preclinical models [28,99,211] and clinical data [16,18,198,212] have revealed Wnt signaling is strongly associated with prostate cancer metastasis. Patients with prostate tumors harboring WNT pathway alterations show a significantly higher metastatic frequency and reduced overall survival, and the frequency of prostate tumors with nuclear β-catenin is significantly increased in patients with metastatic disease relative to those with early locoregional disease [20,205,213]. Nevertheless, how Wnt signaling facilitates prostate cancer metastasis remains unclear. The metastatic cascade is a series of complex processes that are orchestrated by multiple mechanisms (Figure 4), reviewed in [214]. 

Initially, cancer cells breach the basement membrane and migrate away from the primary tumor either as single cells or as a cluster of tumor cells, which is dependent on the tumor cells undergoing EMT [215]. EMT is a reversible biochemical change whereby an epithelial cell adopts a mesenchymal phenotype, allowing transformed cells to dissociate from the primary tumor, invade the local microenvironment and enter the vasculature or lymphatic vessels (known as intravasation) [216]. The interaction between intravasating tumor CTCs and microenvironmental components increases the likelihood of survival and extravasation to distant sites [207]. When CTCs pass through small capillaries, they either cause the vessels to rupture or forces cells to extravasate, potentially by causing necrosis of endothelial cells [208]. As distant sites such as the bone and liver have highly permeable sinusoidal vessels, this increases the likelihood that CTCs will metastasize to these sites [217]. When CTCs reach a secondary metastatic niche, their survival is difficult owing to the harsh microenvironment encountered. For successful colonization, cancer cells need to interact with the host cells of the metastatic site, avoid host immune surveillance, overcome senescence, activate cancer stem cell characteristics, undergo mesenchymal-to-epithelial transition (MET), proliferate, and establish a vascular network [214,218]. Importantly, cancer cells can adopt three phenotypic states; epithelial, mesenchymal or an epithelial-mesenchymal hybrid state. These phenotypes are not static, and cancer cells can transition between all three, depending on multiple factors, including extracellular cues, genetic variants within the disseminated tumor cell, and the extent of colonization [213,219]. If the arriving disseminated tumor cells cannot acclimatize to the harsh metastatic microenvironment, cells may enter a state of dormancy, and it is these dormant tumor cells that are thought to be responsible for delayed relapse [218]. 

Wnt signaling deregulation has been implicated during several stages of the metastatic cascade in multiple human malignancies. For example, while the Wnt pathway is critical for EMT during embryonic development [220], aberrant Wnt signaling can induce an EMT phenotype in cancer cells, promoting migratory and invasiveness capabilities through multiple mechanisms, including the regulation of stem cell activity in prostate cancer [221]. In breast cancer cells, activation of canonical Wnt signaling stabilizes the EMT transcriptional factor snail family transcriptional repressor 2 (SNAI2) by inhibiting GSK3β activity and promoting transcription of EMT-related genes [222]. Interestingly, the Wnt receptor FZD7 is also reported to regulate MET in colon cancer cells [223], highlighting the plasticity of this transition and the complexity of Wnt-mediated EMT and MET during different stages of the metastatic cascade.

Accumulation of genetic alterations within the Wnt signaling pathway has also been shown to promote the invasiveness and metastatic potential of prostate cancer cells, which has been functionally demonstrated in vivo in a number of GEMMs (Table 2). Mechanistically, Wnt/β-catenin signaling has been reported to down-regulate the metastasis suppressor gene kangai 1 (*KAI1*, also known as *CD82*) in prostate cancer cells to potentially facilitate metastatic progression, and *KAI1* expression is frequently decreased in metastatic prostate cancer [224]. Moreover, β-catenin and the REPTIN (RuvB-Like AAA ATPase 2, RUVBL2) chromatin remodeling complex are considered to act in concert with histone deacetylases to antagonize the Tip60 (lysine acetyltransferase 5, KAT5)/PONTIN (RuvB-Like AAA ATPase 1, RUVBL1) co-activator complex, preventing it from binding to the promoter of NF-κB target genes, such as *KAI1*, to suppress transcription [224]. 

The high mortality and morbidity rate associated with advanced prostate cancer is predominantly due to skeletal metastases occurring more commonly in the axial skeleton [225]. To understand the molecular mechanisms driving bone tropism in prostate cancer, gene expression profiles between hindlimb bones (femur, tibia, fibula) that are highly prone to metastases have been studied in comparison with less prone forelimb bones (humerus, radius, and ulna), revealing a Wnt gene signature is upregulated in the hindlimbs compared to the forelimbs [211]. Furthermore, transcription of the Wnt inhibitors *WIF1* and *SOST* (that encode Wnt inhibitory factor-1 and Sclerostin, respectively) was significantly downregulated in the femur relative to the humerus. These data suggest that Wnt signals derived from the femur bone microenvironment could be enhancing prostate cancer bone metastasis. Intriguingly, an enzyme-linked immunosorbent assay (ELISA) detected a two-fold increase in *Wnt5a* in the femur when compared to the humerus and knockdown of *WNT5a* in a bone marrow stromal cell line (HS-5) significantly reduced LNCaP and PC-3 prostate cancer cell invasion in vitro [211]. These findings indicate that non-canonical Wnt signaling may also play a direct role in mediating prostate cancer cell bone tropism. 

Wnt signaling has also been identified as a critical regulator of tumor cell dormancy and reactivation in the bone microenvironment [226]. For instance, osteoblasts derived from neonatal rats can induce quiescence in prostate cancer cells in vitro, and transcriptomic analysis of the osteoblasts detected high expression levels of Wnt ligands (*Wnt5a*, *Wnt5b*, *Wnt11,* and *Wnt16*), suggesting non-canonical Wnt signaling may mediate prostate cancer cell dormancy in bone [142]. Moreover, ELISA analysis revealed a significant increase in *Wnt5a*, Wnt5b, and Wnt16 ligand secretion from the osteoblasts in comparison to PC-3 cells, indicating that osteoblasts are the primary source of non-canonical Wnt ligands [142]. Indeed, *Wnt5a* is reported to induce cell dormancy by reducing Ki67, CyclinD1, and CyclinE1 expression, whilst increasing p21 and p27 expression [142]. Conditioned media from bone marrow-derived mesenchymal stem cells MC3T3-E1 and hFOB1.19 rich in *Wnt5a* can also induce prostate cancer cell dormancy, indicating *Wnt5a* is secreted from a plethora of cell types within the bone microenvironment to promote dormancy of disseminated tumor cells [142]. In vivo experiments using a PC-3 bone metastasis model revealed systemic administration of *Wnt5a* reduces bone metastasis [142]. However, halting *Wnt5a* treatment half way though the experiment increased metastasis, indicating dormancy is *Wnt5a* dependent in this setting [142]. *Wnt5a* is reported to inhibit canonical Wnt/β-catenin signaling from coordinating cell dormancy via the ROR2/SIAH2 signaling axis, which mediates the proteasomal degradation of β-catenin [142]. Furthermore, *Wnt5a*-induced dormant PC-3 cells in xenografts were able to survive in the presence of docetaxel treatment [142]. Collectively these findings suggest Wnt signaling plays a pivotal role in the metastatic spread of prostate cancer cells, and further investigations are needed to clarify at which stage Wnt signaling is required within the metastatic cascade and its role during therapeutic resistance. This will aid the discovery of novel therapeutic targets to inhibit the growth or formation of metastases and inform which patients will benefit from clinical applications of Wnt inhibitors based on disease progression. 

## 5. Targeting the Wnt Cascade to Treat Prostate Cancer

Given the high frequency of oncogenic Wnt signaling observed in prostate cancer, this pathway provides an attractive target for therapeutic intervention. Despite extensive research efforts that have led to the development of a panel of novel agents to inhibit Wnt signaling (reviewed in [227,228,229]), Wnt pathway-directed therapies are yet to be approved to treat prostate cancer. Here, we review several promising therapeutic interventions for targeting the Wnt pathway that has been explored preclinically to treat prostate cancer and/or have shown efficacy against other malignancies (summarized in Table 3). 

### 5.1. Inhibition of β-Catenin

Active canonical Wnt signaling is mainly regulated by the translocation of β-catenin from the cell cytoplasm to the nucleus, where it binds to members of the TCF/LEF transcriptional factor family and CBP to switch on Wnt target gene transcription. Thus, pharmacologically targeting of β-catenin is therapeutically attractive to inhibit downstream canonical Wnt signaling. 

A novel peptidomimetic small molecule inhibitor of β-catenin (CWP232291) is currently in phase 1 trials for acute myeloid leukemia and multiple myeloma [231]. CWP232291 causes endoplasmic reticulum (ER) stress, initiating cell apoptosis ultimately leading to the degradation of β-catenin [20]. Preclinical studies investigating CWP232291 in prostate cancer were performed in a variety of models [230]. In vitro studies revealed that CWP232291 treatment of three metastatic prostate cancer cell lines (PC-3, DU-145, and LNCaP) significantly induces caspase-3 dependent apoptosis and increases the expression of *AXIN2*, a negative regulator of Wnt signaling [230]. CWP232291 treatment also successfully inhibited Wnt signaling by reducing β-catenin activity and the expression of Wnt target genes, such as *MYC* [230]. In AR expressing prostate cancer cell lines, CWP232291 treatment significantly decreased the expression of the AR or AR splice variants (in LNCaP and VCaP cells, respectively), and anti-tumor activity was observed in four primary CRPC patient samples, including a docetaxel-resistant sample [230]. Moreover, CWP232291 treatment inhibits tumor growth in the 22Rv1 subcutaneous CRPC xenograft model in vivo, with a concomitant decrease in β-catenin and increased expression of caspase-3 [230]. These findings indicate CWP232291 could be efficacious against CRPC in the clinic, including CRPC resistance to taxane-based chemotherapy. Importantly, early data from a phase 1 clinical trial exploring CWP232291 treatment for haematological malignancies has reported efficacy with a favorable patient safety profile [231].

In addition to CWP232291, the pharmacological benefit of β-catenin inhibition in prostate cancer using the small molecule inhibitor ICG001 has also been explored preclinically [232]. ICG001 specifically binds to the transcriptional co-activator CBP to diminish the interaction between β-catenin and CBP, causing transcriptional repression of Wnt target genes [21]. ICG001 treatment in combination with enzalutamide synergistically decreases tumor volume relative to monotherapy in the 22Rv1 enzalutamide-resistant CRPC xenograft model [232]. These results indicate that ICG001 can overcome enzalutamide resistance and highlight the efficacious benefit of combining Wnt and androgen/AR pathway targeted therapies to treat CRPC. Interestingly, a second generation β-catenin:CBP antagonist PRI-724, yet to be explored in prostate cancer, is also being clinically investigated for the treatment of β-catenin-activated hepatocellular carcinoma, colorectal, hematological, and pancreatic cancers [233,234]. 

Therapeutic efficacy using the small molecule inhibitor of β-Catenin Responsive Transcription-3 (iCRT3) that disrupts β-catenin and TCF4 interaction has also been observed in prostate cancer [55,235,236]. In human prostate cancer cell lines, iCRT3 treatment is reported to decrease cell viability in vitro associated with reduced transcription of Wnt and AR target genes, and an additive effect was observed when iCRT3 was combined with enzalutamide, supporting the notion that suppressing β-catenin functions may be an effective treatment for enzalutamide resistant CRPC [55,235]. In vivo, iCRT3 treatment has also been shown to reduce tumor burden in the androgen-independent LNCaP-abl (a subline of LNCaP cells) prostate cancer xenograft model [235]. Taken together, these findings indicate that small molecule inhibitors that target β-catenin may show efficacy in patients with prostate cancer, however further research is needed to identify which patient populations are likely to respond and to explore unique avenues for drug delivery to minimize adverse effects associated with systemic down-regulation of Wnt signaling.

### 5.2. Inhibition of Tankyrase1/2

Tankyrases are a family of polymerases that can regulate a number of cellular processes, including proliferation, differentiation, DNA damage repair, and metabolism [251]. Tankyrase1/2 form a distinct subgroup with functional and structural similarities that regulate protein substrates through poly(ADP-ribosyl)ation (PARylation) post-translational modifications and can coordinate a number of cell signaling pathways, including Wnt/β-catenin, YAP, AKT, and NOTCH signaling [251,252]. Tankyrase1/2 can stabilize the Wnt inhibitor AXIN1, resulting in destabilization of the β-catenin destruction complex [253]. G007-LK is a potent tankyrase 1/2 specific small-molecule inhibitor that binds to an adenosine pocket within the poly(ADP-ribosyl)ating (PARP) domain [22]. G007-LK treatment of EPO-GEMM derived cell lines with high Wnt signaling significantly reduced cell growth compared to cells without the Wnt pathway activation [198]. Moreover, G007-LK sensitivity was associated with increased β-catenin phosphorylation and reduced TCF7 reporter activity [198]. In corroboration, in vivo G007-LK treatment of *MPApc*EPO-GEMMs reduced both the primary tumor burden and the incidence of macro-metastatic disease relative to the vehicle controls [198]. These data support the concept that Wnt signaling promotes metastatic progression and indicate that targeting the Wnt pathway with a tankyrase1/2 inhibitor may prove to be efficacious in patients with *APC*-deficient prostate cancer.

### 5.3. Inhibition of Wnt Ligand Secretion

Due to the complexity of signaling in the receiving cells, inhibiting Wnt ligand secretion from the producing cells has been explored to block Wnt signaling. The orally bioavailable small molecule inhibitor LGK974 targets the porcupine enzyme responsible for Wnt ligand secretion and is currently in phase 1 clinical trials as a single agent to explore efficacy in patients with a range of uncurable solid tumors [246]. In a prostate cancer preclinical study, LGK974 treatment is reported to significantly reduce the tumor burden and proliferation in *PBCre4 Ctnnb1^+/^*^Δ*ex3*^ *Pten^+/fl^* mice [18], indicating targeting the Wnt pathway at the level of Wnt ligand secretion is efficacious against aggressive Wnt-driven prostate cancer. In support, LGK974 treatment in the castrated 22Rv1-derived xenograft model also significantly reduced tumor volume (by 86%), attributable to a reduction in Ki-67 and increased cleaved caspase 3 expression [24]. Notably, this study also discovered the requirement of WLS (a regulator of Wnt secretion) in the development of a neuroendocrine phenotype in CRPC, as WLS silencing in both in vitro and in vivo models reduced the proliferative capacity of tumor cells [24]. *WLS* gene expression was observed to increase upon androgen pathway inhibition, which in turn activated PKCδ/ERK signaling to promote NEPC tumor growth, raising the possibility that WLS may be a valuable target for NEPC [24]. Using a Wnt antibody array, several phosphoproteins essential in the non-canonical pathway, such as PKCδ were shown to be downregulated upon WLS silencing [24]. Interestingly, ERK is a downstream effector of PKCδ, and the MAPK/ERK pathway is involved in acquiring a NE phenotype in both prostate cancer and non-small cell lung carcinoma [254]. 

### 5.4. Inhibition of DKK1

DKK1 is overexpressed in advanced prostate, correlating with poor clinical outcome, aggressive tumor growth, high Wnt signaling, and immune evasion [103,151,152,153,154] (Section 2.3.2). Recently, DKK1-high expressing mCRPC biopsies were reported to correlate with high numbers of quiescent natural killer (NK) cells and M2 macrophages and low numbers of activated NK cells and CD8^+^ T-cells [153]. Moreover, the anti-DKK1 blocking antibody DKN-01 can reduce tumor burden in the PC-3 AR/NE negative xenograft model, and the therapeutic benefit was dependent on the presence of NK cells [153]. However, the effect of DKN-01 on the Wnt pathway remains to be determined. Currently, a Phase 1b/2a dose-escalation and dose-expansion study are actively recruiting to explore the efficacy of DKN-01 in DKK1-positive mCRPC, as a monotherapy and in combination with docetaxel (ClinicalTrials.gov identifier: NCT03837353). 

### 5.5. Inhibition of ROR1

Recent work has shown that Wnt/β-catenin signaling not only promotes stem cell maintenance and invasive ability in mCRPC but also potentiates noncanonical *Wnt5a*-ROR1 signaling [255]. The non-canonical Wnt co-receptor ROR1 is elevated in several hematological and solid malignancies, particularly chemo-refractory disease [255,256,257,258,259]. Furthermore, the anti-ROR1 blocking antibody cirmtuzumab has shown efficacy in chronic lymphocytic leukemia (CLL) and mantle cell lymphoma (MCL) trials [247]. A Phase 2 trial to explore Cirmtuzumab is currently being established for patients with mCRPC in combination with docetaxel (ClinicalTrials.gov identifier: NCT05156905). 

### 5.6. Wnt Pathway-Directed Therapies Yet to Be Explored in Prostate Cancer

A number of Wnt-pathway targeting therapeutic agents have been effective against several solid malignancies that remain to be explored in prostate cancer. Here we review a selection of promising Wnt pathway targets that could prove to be efficacious against prostate cancer in the future.

#### 5.6.1. Inhibition of RSPOs

Several studies have identified an oncogenic role for RSPOs in multiple cancer types, reflecting genetic variants, including *RSPO* gene fusions and amplification [163,166]. Accordingly, RSPOs have been explored as a potential therapeutic target. Several anti-RSPO1/2/3 monoclonal antibodies (mAb) have been generated, raised against RSPO1–3. The treatment of several PDX models with an anti-RSPO mAbs has shown these agents to be efficacious in reducing tumor growth in vivo [249]. For example, an anti-RPSO1 mAb has been shown to reduce tumor growth in an ovarian model, treatment with an anti-*RSPO2* mAb reduced tumor growth in both pancreatic and colon cancer models, and an anti-RPSO3 mAb showed significant anti-tumor activity across multiple tumor models, including ovarian, colorectal and non-small cell lung cancers [249]. Anti-RSPO3 mAb treatment is also reported to be effective in two CRC PDX models with *RSPO3* gene fusions (*PTPRK(e1)-RSPO3(e2)*) [250]. 

Interestingly, combining an anti-RSPO3 antibody (OMP-131R10) with paclitaxel also reduced tumor growth and nuclear β-catenin expression in 8 *APC* mutant CRC PDX models when compared to monotherapy [23]. These promising data that span a range of epithelial malignancies indicate that anti-RSPO antibodies may provide a beneficial treatment for prostate cancer that remains to be explored, particularly in patients with *RPSO* alterations (up to 21% of cases, Table 1).

#### 5.6.2. Inhibition of sFRPs

An innovative approach to restoring the expression of sFRPs has recently been identified in studies by exploring demethylating drugs. Epigenetic silencing of *sFRP1* by DNA methylation is linked to taxane resistance in lung adenocarcinoma cell lines and treatment with the demethylating drug 5-azacytidine re-sensitized these cells to taxanes [260]. In addition, 5-aza-2′deoxycytidine (5-aza-dC) is reported to restore *sFRP1* expression in laryngeal cancer cell lines, which led to decreased cell proliferation and colony-forming and sensitized cells to cisplatin in vitro, and tumor growth was impaired in xenografts in vivo [261]. As *SFRP1* downregulation has been linked to epigenetic inactivation in prostate cancer [176,177], further studies to investigate the therapeutic benefit of using DNA methylation drugs for the treatment of advanced prostate cancer is needed.

#### 5.6.3. Inhibition of FZDs

Several approaches have been explored to target the FZD Wnt receptors pharmacologically. The FZD 1/2/5/7/8 blocking antibody vantictumab (OMP-18R5) has shown preclinical efficacy against pancreatic, breast, ovarian, colorectal, and gastric cancers and can synergize with taxanes such as paclitaxel [46,237]. Vantictumab has also been explored in Phase 1b dose-escalation study to treat pancreatic cancer in combination with nab-paclitaxel and gemcitabine in patients with previously untreated metastatic pancreatic adenocarcinoma. However, this study was terminated owing to bone-related safety concerns [238]. 

Additional antibody approaches to inhibit FZDs have also shown some success in various cancer models, including a FZD7 antibody nanoshell conjugates (FZD7-NS) [242] and single-chain variable fragments (scFvs) against FZD7 [243], which were both able to inhibit the growth of triple-negative breast cancer (TNBC) cells. FZD5 antibodies, IgG-2919 and IgG-2921, were able to inhibit the growth of *RNF43* mutant pancreatic ductal adenocarcinoma (PDAC) and CRC cells in vitro and in vivo [239]. Ipafricept (OMP-54F28) is a recombinant fusion protein formed by fusing the immunoglobulin Fc to the FZD cysteine-rich domain (CRD) of FZD8, which competes with FZD8 for WNT ligands [245]. OMP-54F28 was well tolerated in a clinical trial for patients with advanced solid tumors, however, no further trial results have been published to date [245]. More recently, a FZD7-antibody-drug-conjugate has been developed, septuximab vedotin (F7-ADC), which elicits potent tumor regression in ovarian tumor xenografts [240].

Small peptide inhibitors have also been developed to target Wnt signaling at the level of FZD receptors. dFz7-21 is a selective peptide that inhibits FZD1/2/7 by binding to the CRD and preventing the formation of the WNT-FZD-LRP ternary complex, although dFz7-21 has yet to demonstrate any effect on cancer cells [262]. Rhodamine-tagged heptapeptide protein transduction domain for binding DVL (RHPDs) are small interfering peptides that disrupt the binding of FZD7 and the PDZ domain of DVL and were able to inhibit tumor growth in a mouse model of hepatocellular carcinoma (HCC) [244]. A small-molecule compound, SRI37892, which binds to the transmembrane domain of FZD7 is also able to inhibit the growth of breast cancer cells in vitro [241]. As the function of Wnt signaling in regulating the initiation, growth, and progression of prostate cancer is further elucidated, it will be interesting to determine the effect of these FZD inhibitors, or FZD-targeting systems as in the case of septuximab vedotin, in prostate cancer.

## 6. Conclusions

In summary, oncogenic activation of the Wnt pathway plays a significant role in tumor growth, metastasis, and therapeutic resistance. Genomic profiling of prostate cancer patients has identified a broad range of Wnt signaling components that are genetically altered (predominantly *APC* and *CTNNB1*) and highlighted Wnt pathway components as actionable therapeutic targets that may also harbor predictive value for metastatic disease. While a substantial body of work has explored the functional consequence of a handful of Wnt pathway genetic alterations in prostate cancer, several remain to be explored. Furthermore, a deeper understanding of the molecular interplay between the canonical and non-canonical Wnt pathways is also warranted and could uncover novel therapeutic targets.

Although there have been significant developments in modeling prostate cancer over the past decade, several limitations remain. These include the need to expand the small range of models available that fully recapitulate the patient population in terms of disease subtype, metastatic distribution, and the long tail of genetic alterations that could potentiate Wnt signaling in prostate tumors. There is also a lack of models with an intact immune system that reproducibly metastasize from the primary tumor, hindering our understanding of how and when Wnt signaling contributes to metastasis. If we can decipher at what stage of the metastatic cascade Wnt is required and which patients are likely to relapse to current treatment(s) due to deregulated Wnt signaling, this could inform future clinical use of Wnt inhibitors in patients with prostate cancer and improve our management of this lethal disease.

Therapeutically targeting the Wnt pathway remains a current clinical challenge, however, there have been recent advancements of Wnt pathway-directed therapies progressing through clinical trials, such as LGK974. Key questions that remain to be addressed include establishing if prostate cancer patients with a Wnt pathway genetic alteration will respond better to a Wnt pathway-directed therapy such as LGK974, and identifying which disease stage will benefit most from Wnt pathway-directed therapies. 

## Figures and Tables

**Table 1:** Common Wnt pathway genetic variants in prostate cancer.

Gene	Genetic Alteration	Primary Frequency ^1^	Metastasis Frequency ^1^	Predicted Wnt Pathway Response ^2^
*Wnt receptors and co-receptors*				
*FZD3*	Deletion	1.5–12.0%	2–10.0%	Suppressed
*FZD6*	Amplification	3.1–6.3%	10.5–23.2%	Activated
*FZD9*	Amplification	0.8–1.3%	3–5.6%	Activated
*LGR6*	Mutation	0.3–0.4%	1.4–1.8%	Activated
	Amplification	0.0–0.6%	5.7–7.4%	Activated
*LRP5*	Mutation	0.2–0.6%	1.6–2.1%	Activated
	Amplification	1.8–2.1%	7.2%	Activated
*RYK*	Amplification	2.0–2.7%	2.7–7.2%	Activated
*Extracellular regulators of Wnt signaling*				
*DKK4*	Amplification	1.3–2.0%	5.1–6.1%	Wnt/β-cat suppressed
				Wnt/PCP activated
	Deletion	4.3–5.5%	1.6–3.6%	Activated
*RSPO2*	Amplification	2.7–6.5%	7.8–21.0%	Activated
*SFRP1*	Amplification	1.4–1.8%	3.9% - 5.1%	Suppressed
	Deletion	3.2–5.7%	1.2% - 2.0%	Activated
*Intracellular Wnt signaling components*				
*APC*	Deletion	0.9–4.3%	1.1–3.6%	Activated
	Mutation	1.6–2.7%	6.3–7.0%	Activated
*BCL9*	Amplification	0.8–3.7%	2.7–6.8%	Activated
*CTNNB1*	Mutation	1.8–2.6%	4.3–5.4%	Activated
	Amplification	0.2–0.6%	1.8–4.1%	Activated
*DVL3*	Amplification	1.6–2.2%	5.4–8.8%	Activated
*PYGO2*	Amplification	1.8–4.1%	7.5–12.8%	Activated

^1^ Data sourced from the Memorial Sloan Kettering Cancer Centre/Dana-Farber Cancer Institute (MSKCC/DFCI) (primary: *n* = 680, metastatic: *n* = 333) [65], The Cancer Genome Atlas (TCGA) Firehose Legacy (primary: *n* = 492) [66] and the SUC2/PFC International Dream Team (metastatic: *n* = 444) [64] prostate adenocarcinoma datasets, using cBioPortal [68,69], detailed in Tables S1–S4. ^2^ Gray font indicates a hypothetical prediction for Wnt pathway status.

**Table 2:** Summary of GEMMs generated to explore Wnt signaling in prostate cancer.

Model	Prostate Phenotype	Reference
*PBCre4 Apc^fl/fl^*	Hyperplasia (4.5 weeks) and adenocarcinoma (7+ months) with keratinized squamous metaplasia. Castration-resistant.	[181]
*MMTV-LTR Cre Ctnnb1^+/^* ^Δ*ex3*^	Hyperplasia, keratinized squamous metaplasia and PIN (8–12 weeks).	[182,183]
*PBCre4 Ctnnb1^+/^* ^Δ*ex3*^	Hyperplasia (12 weeks) and HG-PIN (6–12 months), castration-resistant.	[184]
	Keratinized squamous metaplasia and PIN (14 weeks), adenocarcinoma (28 weeks) with local invasion (42+ weeks).	[185]
	Metastatic prostate cancer (12–20 months, metastasis = 25% incidence). Late stage castration causes castration-resistant growth.	[18]
*Nkx3.1Cre β-cat^fl/fl^*	Neonatal lethal. Ex vivo E18.5 prostate cultures display impaired budding and branching upon β-catenin deletion.	[186]
*PBCre4 β-cat^fl/fl^*	Normal adult prostate tissue, despite β-catenin deletion.	[187]
*PBCre4 β-cat^fl/fl^ Pten^fl/fl^*	*Pten*-deficient prostate tumor growth unaffected by β-catenin loss (±castration).	[186]
*Nkx3.1-Cre Ctnnb1^+/^* ^Δ*ex3*^	Embryonic lethal. PIN detected when UGS engrafted into the renal capsule.	[184]
	Neonatal lethal. Ex vivo E18.5 prostate cultures show abnormal structures with squamous differentiation (±DHT).	[186]
*Nkx3.1-Cre^ERT2^ Ctnnb1^+/^* ^Δ*ex3*^	Prostate tumor (endpoint, 18–22 months of age). Early castration causes tumor regression.	[18]
	HG-PIN (3 months post-tamoxifen induction), castration-resistant.	[188]
*Nkx3.1-Cre^ERT2^ Apc^fl/fl^*	Prostate hyperplasia (1 month post-induction), HG-PIN (4–10 months post-induction). Castration sensitive.	[188]
*PBCre4 Ctnnb1^+/^* ^Δ*ex3*^ *KRas^+/G12V^*	Locally invasive carcinoma (24 weeks).	[185]
*PBCre4 Ctnnb1^+/^* ^Δ^ *^ex3^* *Pten^+/fl^*	Metastatic prostate cancer (6–12 months, visceral metastasis = 63% incidence), mCRPC growth post-castration.	[18]
*PBCre4 Ctnnb1^+/^* ^Δ*ex3*^ *Pten^fl/fl^*	PIN with focal microinvasive carcinoma (8–14 weeks).	[189]
*PBCre4 Ctnnb1^+/^* ^Δ*ex3*^ *LBP-Tag*	Invasive carcinoma with neuroendocrine differentiation.	[190]
*Nkx3.1-Cre^ERT2^ Ctnnb1^+/^* ^Δ^ *^ex3^* *Pten^+/fl^*	Prostate tumor. Partial castration response.	[18]
*Nkx3.1-Cre^ERT2^ Ctnnb1^+/^* ^Δ*ex3*^ *Pten^fl/fl^*	Prostate tumor. Castration resistant.	[18]
*PBCre4 Ctnnb1^+/^* ^Δ*ex3*^ *Pten^fl/fl^ KRas^+/G12V^*	Diffuse locally invasive carcinoma (12–17 weeks). Lymph node metastasis (10% incidence).	[189]
*PBCre4 Lzts2^fl/fl^ Pten^+/fl^*	HG-PIN (6–8 months), intracystic adenocarcinoma (12 months), and invasive carcinoma (29% incidence, 16 months).	[191]
*PBCre4 Ctnnb1^+/^* ^Δ*ex3*^ *R26hAR^L/+^*	PIN (3 weeks old) and focal microinvasive adenocarcinoma with keratinized squamous metaplasia (5 weeks old), diffuse invasive adenocarcinoma (4+ months).	[192]
*PBCre4 Ctnnb1^+/^*^Δ*ex3*^ Ar^hAR12Q^	LG-PIN (2 months), adenocarcinoma (4 months), invasive carcinoma (6–12 months).	[193]
*PBCre4 Ctnnb1^+/^*^Δ*ex3*^ Ar^hAR21Q^	LG-PIN (2 months), adenocarcinoma (4 months), invasive carcinoma (6–12 months).	[193]
*PBCre4 Ctnnb1^+/^*^Δ*ex3*^ Ar^hAR48Q^	LG-PIN (2 months), HG-PIN (4–6 months) invasive carcinoma (9–12 months).	[193]
*PBCre4 Hi-Myc δ-cat^−/−^*	δ-catenin loss of function accelerates Hi-Myc-driven prostate cancer progression.	[194]
*PBCre4 Apc^fl/fl^ Tgfbr2^fl/fl^*	Invasive adenocarcinoma with keratinized squamous metaplasia (15–28 weeks). Micrometastasis in lumbar lymph node (18% incidence) and lung (12% incidence).	[195]
*PBCre4 Apc^fl/fl^ PB-Hepsin*	Hepsin overexpression and *Apc* deletion cooperate to facilitate invasive prostate cancer progression.	[196]
*Nkx3.1-*Cre^ERT^*Apc^fl^*^/*fl*^*Smad4^fl^*^/*fl*^	Co-deletion of *Apc* and *Smad4* causes invasive prostate cancer progression not observed in single mutants.	[197]
*MPApc* (EPO-GEMM)	Metastatic AR-negative, NE-negative prostate cancer (median survival: 47 days).	[198]
*MPtApc* (EPO-GEMM)	Metastatic prostate cancer.	[198]
*Wnt5a^−/+^ TRAMP AR* ^T877A^	*Wnt5a* depletion reduced NEPC formation and progression.	[199]
*Col1a2-Cre^ERT2^ Ctnnb1^+/^* ^Δ*ex3*^	β-catenin stabilization in stromal cells reduces prostate weight and prostate epithelial cell proliferation.	[200]
*Col1a2-Cre^ERT2^ β-cat^fl/fl^*	β-catenin loss in stromal cells increases prostate weight and prostate epithelial cell proliferation.	[200]

**Table 3:** Selected promising Wnt pathway-directed therapies.

Target	Therapeutic Agent	Description	Reference
β-catenin	CWP232291	Peptidomimetic small molecule inhibitor	[20,230,231]
β-catenin:CBP	ICG001	Small molecule inhibitor	[21,232]
	PRI-724	Small molecule inhibitor	[233,234]
β-catenin:TCF4	iCRT3	Small molecule inhibitor	[55,235,236]
DKK1	DKN-01	Monoclonal antibody	[153]
FZD1/2/5/7/8	Vantictumab (OMP-18R5)	Monoclonal antibody	[46,237,238]
FZD5	IgG-2919 IgG-2921	Monoclonal antibody Monoclonal antibody	[239] [239]
FZD7	Septuximab vedotin (F7-ADC)	Antibody drug conjugate	[240]
	SRI37892	Small molecule inhibitor	[241]
	FZD-NS	Antibody-nanoshell conjugate	[242]
	scFv-I scFv-II	Fusion protein Fusion protein	[243] [243]
FZD7:DVL	RHPDs	Small interfering peptide	[244]
FZD8	Ipafricept (OMP-54F28)	Recombinant fusion protein	[245]
Porcupine	LGK974	Small molecule inhibitor	[18,24,246]
ROR1	Cirmtuzumab	Monoclonal antibody	[247,248]
RSPO3	OMP-131R10	Monoclonal antibody	[23,249,250]
Tankyrase 1/2	G007-LK	Small molecule inhibitor	[22,198]

## Data Availability

Not applicable.

## Supplementary Materials

The following are available online at https://www.mdpi.com/article/10.3390/biom12020309/s1, Figure S1: Frequency of Wnt pathway genetic alterations in primary prostate adenocarcinoma: TCGA Firehose Legacy dataset (*n* = 492 patients/samples); Figure S2: Frequency of Wnt pathway genetic alterations in primary prostate adenocarcinoma: MSKCC/DFCI Nature Genetics 2018 dataset (*n* = 680 samples); Figure S3: Frequency of Wnt pathway genetic alterations in metastatic prostate adenocarcinoma: MSKCC/DFCI dataset (*n* = 333 samples); Figure S4: Frequency of Wnt pathway genetic alterations in metastatic prostate adenocarcinoma: SUC2/PCF IDT 2019 dataset (*n* = 444 samples); Table S1: Frequency of Wnt pathway genetic alterations in primary prostate adenocarcinoma; MSKCC/DFCI dataset, Nature Genetics 2018 (*n* = 680 samples, with mutation and CNA data); Table S2: Frequency of Wnt pathway genetic alterations in primary prostate adenocarcinoma; TCGA Firehose Legacy dataset (*n* = 492 patient/samples with mutation and CNA data); Table S3: Frequency of Wnt pathway genetic alterations in metastatic prostate adenocarcinoma; MSKCC/DFCI dataset, Nature Genetics 2018 (*n* = 333 samples, with mutation and CNA data); Table S4: Frequency of Wnt pathway genetic alterations in metastatic prostate adenocarcinoma; SUC2/PCF IDT dataset, PNAS 2019 (*n* = 444 samples, with mutation and CNA data); Table S5: File Raw Data.; Table S6: File Raw Data.

## Abbreviations

ADT	Androgen deprivation therapy
AF-1	Activation function-1
APC	Adenomatous polyposis coli
AR	Androgen receptor
AREs	Androgen response elements
AXIN1/2	Axis inhibitior 1/2
β-TrCP	Beta-transducin repeat containing E3 ubiquitin protein ligase
BCL9	B-cell lymphoma 9
BRCA1/2	Breast cancer gene 1/2
CAM	Chick chorioallantoic membrane
CBP	CREB binding protein
CCND1	Cyclin D1
cfDNA	Cell-free DNA
CK1α	Casein kinase 1α
CK1δ/ε	Casein kinase 1 delta/epsilon
CKAP4	Cytoskeleton-associated protein 4
CRC	Colorectal cancer
CRD	Cysteine-rich domain
CRPC	Castration-resistant prostate cancer
CTCs	Circulating tumor cells
CTNNB1	Catenin Beta 1
DAG	1,2 diacylglycerol
DHT	Dihydrotestosterone
DKK	Dickkopf
DVL	Disheveled
ELISA	Enzyme-linked immunosorbent assay
EMT	Epithelial-to-mesenchymal transition
FZD	Frizzled
FACS	Fluorescence-activated cell sorting
FAP	Familial adenomatous polyposis coli
GEMMs	Genetically engineered mouse models
GPCRs	G-protein coupled receptors
GR	Glucocorticoid receptor
GSEA	Gene set enrichment analysis
GSK3β	Glycogen synthase-3 beta
H3K4me	Methylated histone H3, lysine 4
HCC	Hepatocellular carcinoma
HG-PIN	High-grade prostate intraepithelial neoplasia
HR	Hazard ratio
IGF1R	Insulin-like growth factor 1 receptor
IHC	Immunohistochemistry
IP3	Inositol 1,4,5-triphosphate
JNK	Jun-N-terminal kinase
KAI1	Kangai 1 (CD82)
LATS2	Large tumor suppressor kinase 2
LBD	Ligand-binding domain
LG-PIN	Low-grade prostate intraepithelial neoplasia
LGR4/5/6	Leucine rich repeat containing G protein-coupled receptor 4/5/6
LRP5/6	Low-density lipoprotein receptor-5/6
LZTS2	Leucine zipper tumor suppressor 2
mAb	Monoclonal antibody
MAPK	Mitogen-activated protein kinase
MCL	Mantle cell lymphoma
mCRPC	Metastatic castration resistant prostate cancer
mHSPC	Metastatic hormone sensitive prostate cancer
MET	Mesenchymal-to-epithelial transition
MMP2	Matrix metalloproteinase-2
MYC	MYC Proto-Oncogene, BHLH Transcription Factor
NEPC	Neuroendocrine prostate cancer
NFAT	Nuclear factor of activated T-cells
NF-κB	Nuclear factor kappa B
NK	Natural killer
NSCLC	Non-small cell lung cancer
OS	Overall survival
PARP	Poly(ADP-ribose) polymerase
PARylation	Poly(ADP-ribosyl)ation
PCP	Planar cell polarity
PDO	Patient-derived organoid
PDX	Patient-derived xenograft
PDX-Os	Patient-derived xenograft organoids
PI3K	Phosphoinositide 3-kinase
PIN	Prostate intraepithelial neoplasia
PKC	Protein kinase C
PLC	Phospholipase C
polyQ	Polyglutamine
PONTIN	RuvB-Like AAA ATPase 1, RUVBL1
PR	Progesterone receptor
PSA	Prostate specific antigen
PTEN	Phosphatase and tensin homologue deleted on chromosome 10
PYGO1/2	Pygopus Family PHD Finger
RAC	Rac Family Small GTPase
RAS	Rat sarcoma virus
REIC	Reduced expression in immortalized cells
REPTIN	RuvB-Like AAA ATPase 2, RUVBL2
RhoA	Ras homolog family member A
RNF43	Ring finger protein 43
ROR2	Receptor tyrosine kinase-like orphan receptor 2
ROCK1	Rho associated protein kinase
R-SPO	R-Spondin
RYK	Receptor tyrosine kinase
sFRPs	Secreted frizzled-related proteins
SNAI2	Snail family transcriptional repressor 2
SOST	Sclerostin
TGFB2	TGFβ type II receptor
TCF/LEF	T-cell factor/lymphoid enhancer factor
Tip60	Lysine acetyltransferase 5, KAT5
TMPRSS2-ERG	Transmembrane serine protease 2–erythroblast transformation specific (ETS) Transcription Factor ERG fusion protein
TNBC	Triple-negative breast cancer
t-NEPC	Treatment-induced neuroendocrine prostate cancer
UGS	Urogenital sinus
VANGL1/2	Van gogh-like protein 1/2
WIF1	Wnt inhibitory factor 1
WLS	Wntless
YAP	Yes-associated protein
ZNRF3	Zinc and ring finger 3
