# Supplementary material for: Exploring the Wnt Pathway as a Therapeutic Target for Prostate Cancer
Source: Biomolecules. 2022 Feb 15;12(2):309. doi: 10.3390/biom12020309 (PMC8869457; doi:10.3390/biom12020309)
Supplement: Supplementary file 1 [file biomolecules-12-00309-s001.zip › Table S1.pdf]

Table S1: Frequency of Wnt pathway genetic alterations in primary prostate adenocarcinoma; MSKCC/DFCI dataset, Nature Genetics 2018 (n = 680 samples, with mutation and CNA data).

Gene (protein)	Mutation (%)	Amplification (%)	Deep deletion (%)	Multiple alterations (%)	Total (%)
**Wnt receptors/co-receptors**					
FZD1	0.15	2.94	0.15	0	3.24
FZD2	0.29	0	0	0	0.29
FZD3	0.15	0.44	1.47	0	2.06
FZD4	0.44	0.74	0.29	0	1.47
FZD5	0.44	1.03	0.29	0	1.76
FZD6	0.44	3.09	0	0	3.53
FZD7	0.29	0.29	0.59	0	1.18
FZD8	0	0.15	0.29	0	0.44
FZD9	0.59	1.32	0.15	0	2.06
FZD10	0.29	0	0	0	0.29
LGR4	0.15	1.32	0	0	1.47
LGR5	0.29	0	0	0	0.29
LGR6	0.29	0.59	0.29	0	1.18
LRP5	0.15	2.06	0.15	0	2.35
LRP6	0.29	0	0	0	0.29
RNF43	0.88	0	1.18	0	2.06
ROR1	0.15	0.15	1.18	0	1.47
ROR2	0.44	0.29	0.59	0	1.32
RYK	0.29	2.65	0	0	2.94
VANGL1	0	0.88	0.44	0	1.32
VANGL2	0.29	0.88	2.06	0	3.24
ZNRF3	0.44	0	0	0	0.44
**Extracellular regulators of Wnt signalling**					
DKK1	0.44	0.88	0.88	0	2.21
DKK2	0.29	0.74	0.29	0	1.32
DKK3	0	1.47	0.29	0	1.76
DKK4	0	1.32	4.26	0	5.59
RSPO1	0.29	0.29	0.44	0	1.03
RSPO2	0.44	2.65	0.44	0	3.53
RSPO3	0.15	0.74	1.18	0	2.06
RSPO4	0	0	0	0	0
SFRP1	0	1.47	3.24	0	4.71
SFRP2	0.59	1.47	0.15	0	2.21
SFRP3 (FRZB)	0	0.44	2.5	0	2.94
SFRP4	0.15	1.47	0.15	0	1.76
SFRP5	0.15	0.59	1.91	0	2.65
WIF1	0.15	0	0	0	0.15
WNT1	0.15	0	0	0	0
WNT2	0.74	2.79	0.15	0	0
WNT2B	0.15	0.88	0.44	0	1.47

WNT3	0.29	0	0	0	0.29
WNT3A	0.15	0.15	3.82	0	4.12
WNT4	0.15	0.15	1.32	0	1.62
WNT5A	0.29	0.74	0.59	0	1.62
WNT5B	0.15	0	0	0	0.15
WNT6	0.44	0.29	0.88	0	1.62
WNT7A	0.29	1.03	0.44	0	1.76
WNT7B	0.15	0	0	0	0.15
WNT8A	0	0.59	0.74	0	1.32
WNT8B	0	0.74	1.62	0	2.35
WNT9A	0.29	0.29	3.82	0	4.41
WNT9B	0	0	0	0	0
WNT10A	0	0.29	0.88	0	1.18
WNT10B	0	0	0	0	0
WNT11	0.15	0.44	0.15	0	0.74
WNT16	0	2.5	0.44	0	2.94
**Intracellular Wnt signalling components**					
APC	2.65	0	0.88	0	2.65
AXIN1	0.15	0	6.62	0	6.76
AXIN2	0.44	0	0.29	0	0.74
BCL9	0.59	3.68	0.29	0	4.56
CTNNB1	1.76	0.59	0	0	2.35
DVL1	0.15	0.74	6.76	0	7.65
DVL2	0.59	0	0	0	0.59
DVL3	0.29	2.21	1.18	0	3.68
GSK3B	0.44	0.44	0.15	0	1.03
PORCN	0.15	0	0	0	0.15
PYGO1	0.15	0	0	0	0.15
PYGO2	0	4.12	0.15	0	4.26
TCF3	0.15	0	0	0	0.15
TCF4	0.15	0	0	0	0.15
TCF7	0.15	0.44	0.88	0	1.47
