# Supplementary material for: Exploring the Wnt Pathway as a Therapeutic Target for Prostate Cancer
Source: Biomolecules. 2022 Feb 15;12(2):309. doi: 10.3390/biom12020309 (PMC8869457; doi:10.3390/biom12020309)
Supplement: Supplementary file 1 [file biomolecules-12-00309-s001.zip › Table S2.pdf]

Table S2: Frequency of Wnt pathway genetic alterations in primary prostate adenocarcinoma; TCGA Firehose Legacy dataset (n = 492 patient/samples with mutation and CNA data).

Gene (protein)	Mutation (%)	Amplification (%)	Deep deletion (%)	Multiple alterations (%)	Total (%)
**Wnt receptors/co-receptors**					
FZD1	0.41	0.81	0.2	0	1.42
FZD2	0.2	0.2	4.88	0	5.28
FZD3	0	0.2	11.99	0	12.2
FZD4	0.2	0.41	0	0	0.61
FZD5	0.61	0	0.2	0	0.81
FZD6	0.61	6.3	0	0	6.91
FZD7	0.41	0	1.22	0	1.63
FZD8	0	0.2	0.41	0	0.61
FZD9	0.41	0.81	0	0	1.22
FZD10	0.61	0.41	1.22	0	2.24
LGR4	0.2	0.41	0	0	0.61
LGR5	0.41	0.81	0.2	0	1.42
LGR6	0.41	0	0.41	0	0.81
LRP5	0.61	1.83	0.2	0	2.64
LRP6	0.41	0	4.88	0	5.28
RNF43	1.02	0.61	0.81	0.2	2.64
ROR1	0.2	0	2.64	0	2.85
ROR2	1.02	0.61	0.41	0	2.03
RYK	0.41	2.03	0.2	0	2.64
VANGL1	0	0	0.61	0	0.61
VANGL2	0.61	0.41	0.81	0	1.83
ZNRF3	0.2	0.2	2.03	0	2.44
**Extracellular regulators of Wnt signalling**					
DKK1	0.61	0	1.83	0	2.44
DKK2	0.41	0	0.41	0	0.81
DKK3	0	0.2	0.2	0	0.41
DKK4	0	2.03	5.49	0	7.52
RSPO1	0.41	0	0.41	0	0.81
RSPO2	0.61	6.5	1.02	0	8.13
RSPO3	0.2	0	1.42	0	1.63
RSPO4	0	0.2	0.61	0	0.81
SFRP1	0.2	1.83	5.69	0	7.72
SFRP2	0.61	0	0.2	0	0.81
SFRP3 (FRZB)	0	0.2	2.24	0	2.44
SFRP4	0	0.41	0.2	0	0.61
SFRP5	0.2	0.41	2.64	0	3.25
WIF1	0.41	1.02	0.2	0	1.63
WNT1	0.2	0.41	0	0	0.61
WNT2	0.41	0.2	1.42	0	2.03
WNT2B	0.2	0	0.61	0	0.81

WNT3	0	0.61	2.03	0	2.64
WNT3A	0.2	0	2.85	0	3.05
WNT4	0.2	0.2	0.61	0	1.02
WNT5A	0.41	0.2	0.81	0	1.42
WNT5B	0.2	0.2	0.81	0	1.22
WNT6	1.02	0	0.81	0	1.83
WNT7A	0.2	0.2	0.61	0	1.02
WNT7B	0	0	1.42	0	1.42
WNT8A	0	0.41	0.61	0	1.02
WNT8B	0	0.61	2.44	0	3.05
WNT9A	0.41	0	2.85	0	0
WNT9B	0	0.61	2.03	0	2.64
WNT10A	0	0	0.81	0	0.81
WNT10B	0.2	0.41	0	0	0.61
WNT11	0	0.61	0	0	0.61
WNT16	0	1.02	0.61	0	1.63
**Intracellular Wnt signalling components**					
APC	1.63	0.2	4.27	0.41	6.5
AXIN1	0.2	0.81	0.81	0	1.83
AXIN2	0.81	1.02	0	0	1.83
BCL9	0.81	0.81	0	0	1.63
CTNNB1	2.64	0.2	0.2	0	3.05
DVL1	0.2	0.41	0.2	0	0.81
DVL2	0.41	0	6.3	0	6.71
DVL3	0.2	1.63	1.02	0	2.85
GSK3B	0.2	0.81	0.81	0	1.83
PORCN	0	0.61	0	0	0.61
PYGO1	0.2	0	1.22	0	1.42
PYGO2	0	1.83	0	0	1.83
TCF3	0.41	0	0.61	0	1.02
TCF4	0.2	0	3.46	0	3.66
TCF7	02	02	1.42	0	1.83
