# Supplementary material for: Exploring the Wnt Pathway as a Therapeutic Target for Prostate Cancer
Source: Biomolecules. 2022 Feb 15;12(2):309. doi: 10.3390/biom12020309 (PMC8869457; doi:10.3390/biom12020309)
Supplement: Supplementary file 1 [file biomolecules-12-00309-s001.zip › Table S3.pdf]

Table S3: Frequency of Wnt pathway genetic alterations in metastatic prostate adenocarcinoma; MSKCC/DFCI dataset, Nature Genetics 2018 (n = 333 samples, with mutation and CNA data).

Gene (protein)	Mutation (%)	Amplification (%)	Deep deletion (%)	Multiple alterations (%)	Total (%)
**Wnt receptors/co-receptors**					
FZD1	1.2	3.9	0	0	5.11
FZD2	1.5	0	0	0	1.5
FZD3	0.6	0.9	2.1	0	3.6
FZD4	0.6	1.5	0.9	0.3	3.3
FZD5	1.2	1.8	0.3	0	3.3
FZD6	0.3	10.51	0	0	10.81
FZD7	0.6	1.2	0.3	0	2.1
FZD8	1.2	1.8	0.9	0	3.9
FZD9	0.9	3	1.2	0	5.11
FZD10	2.1	0	0	0	0
LGR4	0.6	2.1	0	0	0
LGR5	0.9	0	0	0	0.9
LGR6	1.8	5.71	0.3	0.6	8.41
LRP5	2.1	7.21	1.5	0	10.81
LRP6	1.5	0	0	0	1.5
RNF43	1.8	2.1	0.3	0	4.2
ROR1	0.6	0.6	2.1	0	3.3
ROR2	2.4	1.5	0	0	3.9
RYK	0.3	2.7	0.3	0	3.3
VANGL1	0.6	1.5	0.9	0	3
VANGL2	0.3	1.2	0.3	0	1.8
ZNRF3	1.8	0	0	0	1.8
**Extracellular regulators of Wnt signalling**					
DKK1	0	3.3	1.2	0	4.5
DKK2	0.6	1.8	0	0	2.4
DKK3	0	0.6	0.6	0	1.2
DKK4	0	5.11	3.6	0	8.71
RSPO1	0.3	0.6	0.3	0	1.2
RSPO2	0	7.81	0.6	0	8.41
RSPO3	0.3	1.8	0.9	0	3.0
RSPO4	0.3	0	0	0	0.3
SFRP1	0.3	3.9	1.2	0	5.41
SFRP2	0.3	2.7	0.9	0	3.9
SFRP3 (FRZB)	0	1.2	1.5	0	2.7
SFRP4	0	1.8	0.9	0	2.7
SFRP5	0	2.1	2.4	0	4.5
WIF1	0	0	0	0	0
WNT1	0	0	0	0	0
WNT2	1.5	2.4	0.3	0	4.2
WNT2B	0.3	0.9	0.6	0	1.8

WNT3	0	0	0	0	0
WNT3A	0.6	0.6	0.6	0	1.8
WNT4	0.3	0.9	1.2	0	2.4
WNT5A	0.9	0.3	0.3	0	1.5
WNT5B	0.9	0	0	0	0
WNT6	0.6	1.2	0.9	0	2.7
WNT7A	0.3	1.2	0.3	0	1.8
WNT7B	0	0	0	0	0
WNT8A	0.3	1.5	0.9	0	2.7
WNT8B	0	0.6	1.8	0.3	2.7
WNT9A	0.3	0.6	0.6	0	1.5
WNT9B	0.3	0	0	0	0.3
WNT10A	0	1.5	0.9	0	2.4
WNT10B	1.8	0	0	0	1.8
WNT11	0.3	0.3	0.6	0	1.2
WNT16	1.5	2.1	0.3	0	3.9
**Intracellular Wnt signalling components**					
APC	6.31	0	3.6	0	9.91
AXIN1	1.5	0	2.4	0	3.9
AXIN2	0.6	0	0.6	0	1.2
BCL9	1.8	2.7	0.3	0	4.8
CTNNB1	5.41	1.8	0	0	7.21
DVL1	0.6	0.9	2.1	0	3.6
DVL2	0.9	0	0	0	0.9
DVL3	0.9	5.41	0.3	0	6.61
GSK3B	0	1.2	0.3	0	1.5
PORCN	0.6	0	0	0	0.6
PYGO1	0.3	0	0	0	0.3
PYGO2	0	7.51	0	0	7.51
TCF3	0.9	0	0	0	0.9
TCF4	1.5	0	0	0	1.5
TCF7	0.3	0.9	0.6	0	1.8
