# Supplementary material for: Exploring the Wnt Pathway as a Therapeutic Target for Prostate Cancer
Source: Biomolecules. 2022 Feb 15;12(2):309. doi: 10.3390/biom12020309 (PMC8869457; doi:10.3390/biom12020309)
Supplement: Supplementary file 1 [file biomolecules-12-00309-s001.zip › Table S4.pdf]

Table S4: Frequency of Wnt pathway genetic alterations in metastatic prostate adenocarcinoma; SUC2/PCF IDT dataset, PNAS 2019 (n = 444 samples, with mutation and CNA data).

Gene (protein)	Mutation (%)	Amplification (%)	Deep deletion (%)	Multiple alterations (%)	Total (%)
**Wnt receptors/co-receptors**					
FZD1	0.68	4.5	0.23	0.23	5.63
FZD2	1.35	2.03	0.68	0	4.05
FZD3	0.68	1.58	10.36	0	12.61
FZD4	0.45	1.58	0	0	2.03
FZD5	0.45	4.28	0	0	4.73
FZD6	0.23	23.2	0	0	23.42
FZD7	0.45	2.25	0.23	0	2.93
FZD8	1.13	1.13	1.13	0	3.38
FZD9	0.68	5.63	0	0	6.31
FZD10	1.58	3.83	0.9	0	6.31
LGR4	0.23	2.48	0.45	0	3.15
LGR5	0.23	4.95	0.23	0	5.41
LGR6	1.35	7.43	0	0.45	9.23
LRP5	1.58	7.21	0.23	0	9.01
LRP6	1.13	2.7	4.28	0	8.11
RNF43	2.48	4.05	0.68	0	7.21
ROR1	0.68	0.45	1.13	0	2.25
ROR2	1.35	4.05	0	0	5.41
RYK	0	7.21	0	0	7.21
VANGL1	0.23	1.8	0	0	2.03
VANGL2	0.23	5.86	0	0	6.08
ZNRF3	1.8	1.35	1.35	0	4.5
**Extracellular regulators of Wnt signalling**					
DKK1	0	4.05	1.58	0	5.63
DKK2	0.68	1.35	0.68	0	2.7
DKK3	0.23	1.35	0	0	1.58
DKK4	0	6.08	1.58	0.23	7.88
RSPO1	0.45	0.23	0.23	0	0.9
RSPO2	0	20.95	0.23	0	21.17
RSPO3	0.45	1.35	0.68	0	2.48
RSPO4	0.23	2.03	0.9	0	3.15
SFRP1	0.23	5.18	2.03	0	7.43
SFRP2	0.23	2.93	0.68	0	3.83
SFRP3 (FRZB)	0	2.03	0.45	0	2.48
SFRP4	0	4.05	0	0	4.05
SFRP5	0	1.35	2.25	0	3.6
WIF1	0.23	2.03	0	0	2.25
WNT1	0.23	1.13	0	0	1.35
WNT2	1.8	3.38	1.35	0.23	6.76
WNT2B	0.68	1.13	0.45	0	2.25

WNT3	0	3.83	0.45	0	4.28
WNT3A	0.45	3.38	1.8	0	5.63
WNT4	0.23	0.23	0	0	0.45
WNT5A	0.9	0.45	0.45	0	1.8
WNT5B	0.68	3.38	0.45	0	4.5
WNT6	0.9	2.03	1.13	0	4.05
WNT7A	0.45	2.93	0.23	0	3.6
WNT7B	0	0.9	2.48	0	3.38
WNT8A	0.23	2.03	0.45	0	2.7
WNT8B	0	1.35	3.15	0	4.5
WNT9A	0.45	3.15	1.58	0	5.18
WNT9B	0.45	3.83	0.45	0	4.73
WNT10A	0	2.03	1.35	0	3.38
WNT10B	1.35	0.9	0	0	2.25
WNT11	0.45	2.48	0.23	0	3.15
WNT16	1.13	3.38	0.45	0	4.95
**Intracellular Wnt signalling components**					
APC	6.98	0	1.13	0	8.11
AXIN1	1.13	6.31	0.9	0	8.33
AXIN2	0.68	4.5	0.45	0	5.63
BCL9	1.8	6.76	0	0	8.56
CTNNB1	4.28	4.05	0.23	0	8.56
DVL1	0.23	1.58	1.13	0	2.93
DVL2	0.68	1.13	4.28	0	6.08
DVL3	0.68	8.78	0	0	9.46
GSK3B	0	3.15	0.9	0	4.05
PORCN	0.45	9.01	0	0	9.46
PYGO1	0	0.68	0.68	0	1.35
PYGO2	0	12.84	0	0	12.84
TCF3	0.68	1.35	2.25	0	4.28
TCF4	0.68	0.9	1.58	0	3.15
TCF7	0.68	1.13	1.13	0	2.93
