# Supplementary material for: Exploring the Wnt Pathway as a Therapeutic Target for Prostate Cancer
Source: Biomolecules. 2022 Feb 15;12(2):309. doi: 10.3390/biom12020309 (PMC8869457; doi:10.3390/biom12020309)

Figure S2: Frequency of Wnt pathway genetic alterations in primary prostate adenocarcinoma: MSKCC/DFCI Nature Genetics 2018 dataset (n = 680 samples).

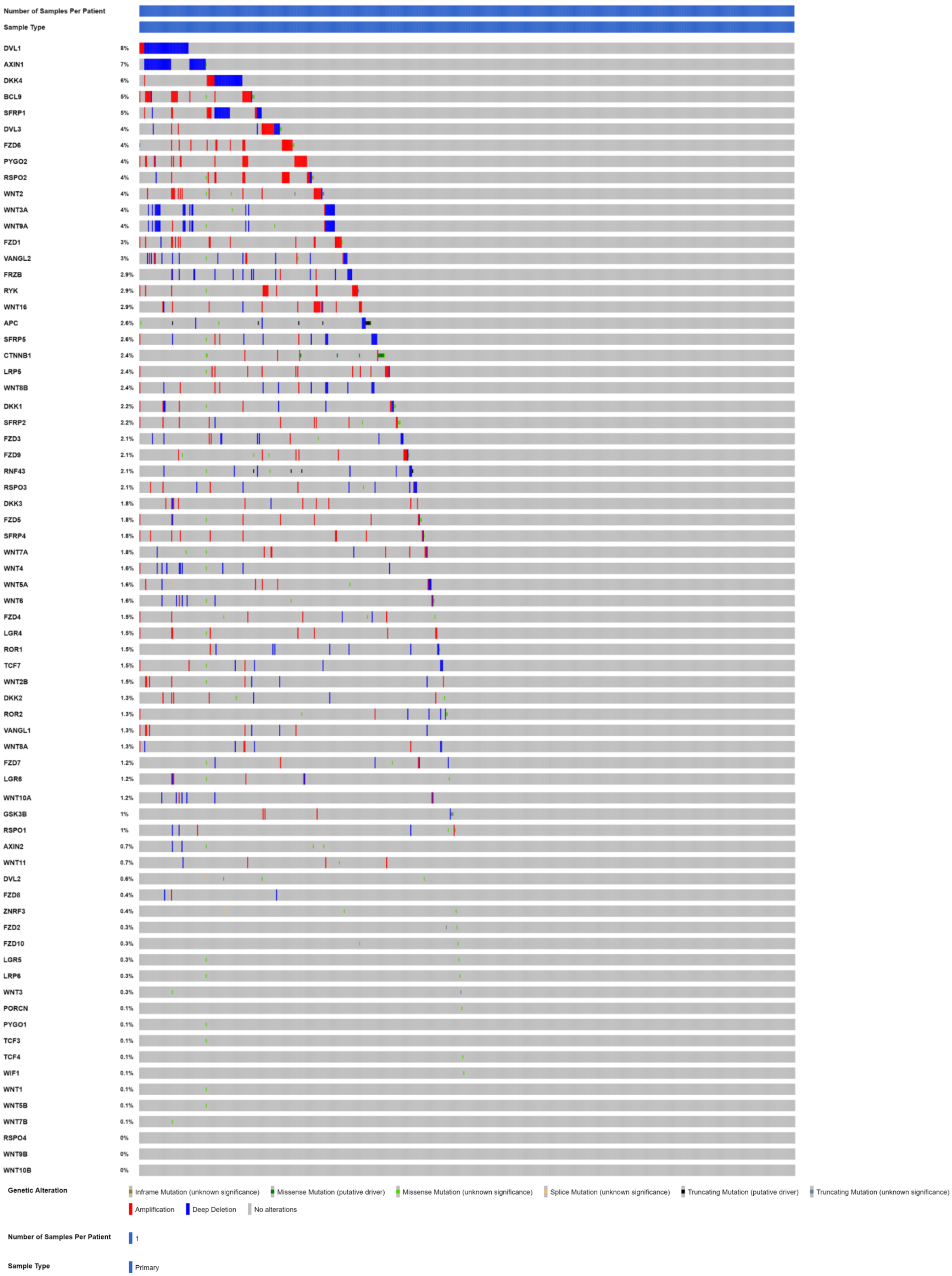

Supplement: Supplementary file 1 [file biomolecules-12-00309-s001.zip › Figure S2.pdf]
