# Supplementary material for: Exploring the Wnt Pathway as a Therapeutic Target for Prostate Cancer
Source: Biomolecules. 2022 Feb 15;12(2):309. doi: 10.3390/biom12020309 (PMC8869457; doi:10.3390/biom12020309)

Figure S3: Frequency of Wnt pathway genetic alterations in metastatic prostate adenocarcinoma: MSKCC/DFCI dataset (n = 333 samples).

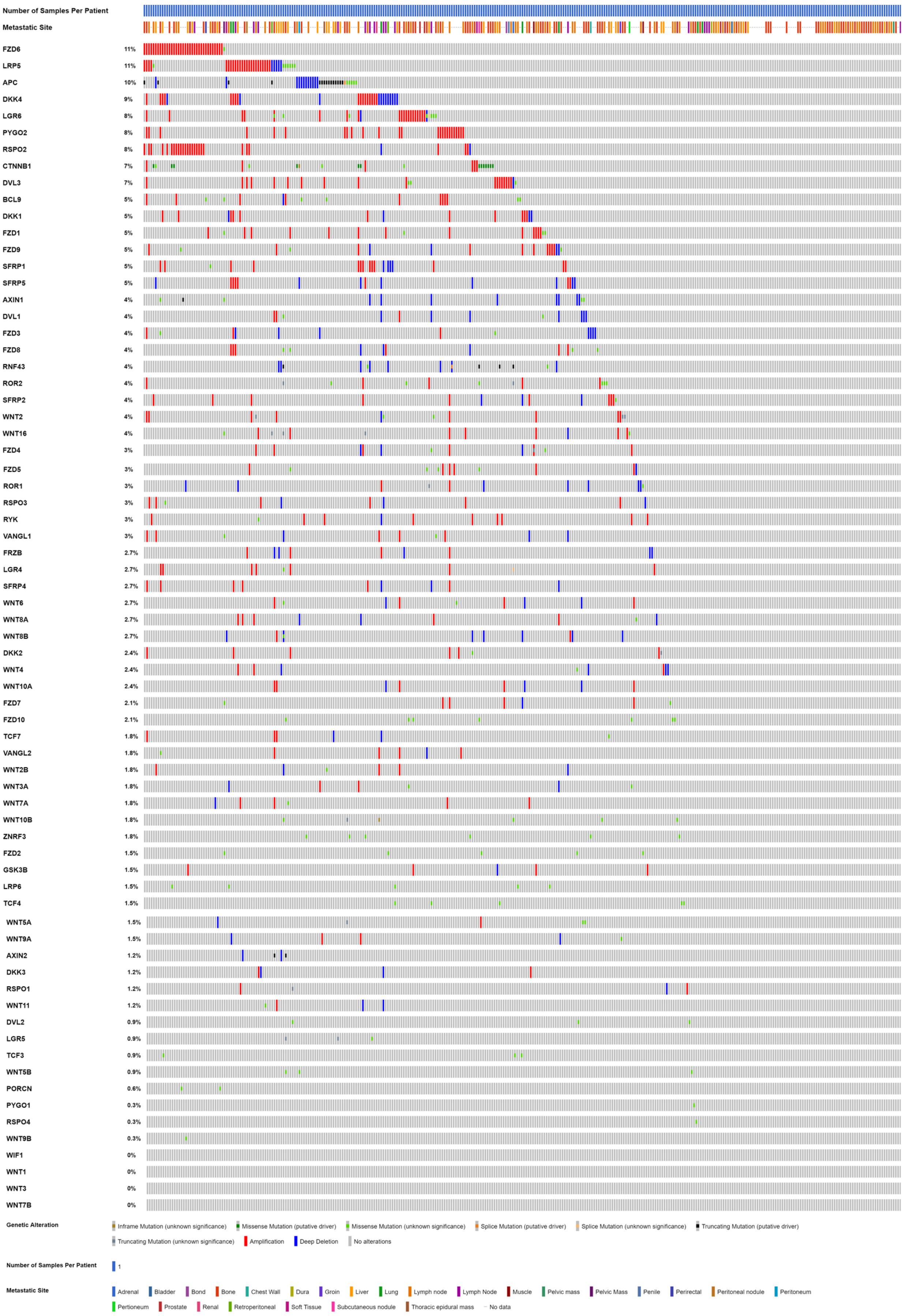

Supplement: Supplementary file 1 [file biomolecules-12-00309-s001.zip › Figure S3.pdf]
